# A longitudinal assessment of host-microbe-parasite interactions resolves the zebrafish gut microbiome’s link to *Pseudocapillaria tomentosa* infection and pathology

**DOI:** 10.1186/s40168-019-0622-9

**Published:** 2019-01-24

**Authors:** Christopher A. Gaulke, Mauricio L. Martins, Virginia G. Watral, Ian R. Humphreys, Sean T. Spagnoli, Michael L. Kent, Thomas J. Sharpton

**Affiliations:** 1 Department of Microbiology, Oregon State University, Corvallis, OR 97330 USA; 2 AQUOS—Aquatic Organisms Health Laboratory, Aquaculture Department, Federal University of Santa Catarina, Florianopolis, SC Brazil; 3 Department of Biomedical Sciences, Oregon State University, Corvallis, OR USA; 4 Department of Statistics, Oregon State University, Corvallis, OR 97330 USA

**Keywords:** Zebrafish, Microbiome, Intestine, Parasitism, Nematode, *Pseudocapillaria tomentosa*

## Abstract

**Background:**

Helminth parasites represent a significant threat to the health of human and animal populations, and there is a growing need for tools to treat, diagnose, and prevent these infections. Recent work has turned to the gut microbiome as a utilitarian agent in this regard; components of the microbiome may interact with parasites to influence their success in the gut, meaning that the microbiome may encode new anthelmintic drugs. Moreover, parasite infections may restructure the microbiome’s composition in consistent ways, implying that the microbiome may be useful for diagnosing infection. The innovation of these utilities requires foundational knowledge about how parasitic infection, as well as its ultimate success in the gut and impact on the host, relates to the gut microbiome. In particular, we currently possess limited insight into how the microbiome, host pathology, and parasite burden covary during infection. Identifying interactions between these parameters may uncover novel putative methods of disrupting parasite success.

**Results:**

To identify interactions between parasite success and the microbiome, we quantified longitudinal associations between an intestinal helminth of zebrafish, *Pseudocapillaria tomentosa*, and the gut microbiome in 210 4-month-old 5D line zebrafish. Parasite burden and parasite-associated pathology varied in severity throughout the experiment in parasite-exposed fish, with intestinal pathologic changes becoming severe at late time points. Parasite exposure, burden, and intestinal lesions were correlated with gut microbial diversity. Robust generalized linear regression identified several individual taxa whose abundance predicted parasite burden, suggesting that gut microbiota may influence *P. tomentosa* success. Numerous associations between taxon abundance, burden, and gut pathologic changes were also observed, indicating that the magnitude of microbiome disruption during infection varies with infection severity. Finally, a random forest classifier accurately predicted a fish’s exposure to the parasite based on the abundance of gut phylotypes, which underscores the potential for using the gut microbiome to diagnose intestinal parasite infection.

**Conclusions:**

These experiments demonstrate that *P. tomentosa* infection disrupts zebrafish gut microbiome composition and identifies potential interactions between the gut microbiota and parasite success. The microbiome may also provide a diagnostic that would enable non-destructive passive sampling for *P. tomentosa* and other intestinal pathogens in zebrafish facilities.

**Electronic supplementary material:**

The online version of this article (10.1186/s40168-019-0622-9) contains supplementary material, which is available to authorized users.

## Introduction

Intestinal helminth infections present a major global health concern [1, 2]. Over one billion people suffer helminth infections [1, 2], which often manifest developmental, cognitive, nutritional, and gastrointestinal defects [3–5]. While safe and effective drugs can treat helminth infection, the rapid rise of anthelmintic drug resistance [6] necessitates development of new strategies for parasite control. Hope for such strategies springs from the observation that parasite-associated morbidity unevenly distributes across populations [5], which indicates that personalized factors impact infection outcome [7–9]. Elucidating these factors may uncover new methods or drug leads to effectively treat, predict, and ultimately prevent helminthic infection.

A growing body of evidence points to the gut microbiome as an agent that interacts with intestinal parasites to influence their growth or mediate their physiological effect on an individual. For example, helminth-infected humans [10, 11] and mice [12] harbor gut microbiomes with significantly different structures and diversity than uninfected controls. Additionally, studies in mice have found that helminth infection perturbs specific gut bacteria to yield alterations in host immune status [13]. Other work has shown that the gut microbiome acts as an innate immune barrier to intestinal infection [14]. Despite this prior research, our insight into how the gut microbiome, individual gut microbiota, or microbial metabolites might contribute to parasite success in the gut remains limited. Importantly, the extent to which microbiome composition or diversity associates with parasite burden and intestinal pathology across the length of infection remains unclear. Understanding these dynamics will enhance our ability to resolve specific microbial taxa that may influence or respond to parasite exposure and identify putative microbial therapeutics and diagnostics. For example, as parasite burden and tissue damage are unlikely to be uniformly distributed throughout infection, longitudinal analyses may allow identification of altered microbiota abundance that corresponds to increases or decreases in the worm abundance or intestinal pathology.

Here, we longitudinally assessed the gut microbiome of zebrafish (*Danio rerio*) infected with *Pseudocapillaria tomentosa*, an important cause of disease in zebrafish facilities [15], to clarify how the gut microbiome varies in accordance with intestinal parasitic infection dynamics. *Pseudocapillaria tomentosa* is a capillarid nematode, closely relates to whipworm species that infect humans and other mammals [16], and preferentially infects the guts of fish to induce intestinal inflammation, tissue damage, and epithelial hyperplasia [17]. Fish infected with *P. tomentosa* often appear emaciated and lethargic, although cryptic subclinical infections have also been reported [15]. We monitored the gut microbiome, parasite burden, and tissue damage in *P. tomentosa*-infected fish over 12 weeks of infection. We found that *P. tomentosa* infection associates with inflammation, hyperplasia, and weight loss over the course of the experiment. Moreover, *P. tomentosa* exposure alters microbial community diversity. Additionally, regression models revealed that parasite burden and gut lesions link to the abundance of several individual microbiota, indicating that these taxa may influence infection outcomes or are sensitive to infection. Finally, using machine learning, we demonstrate that the gut microbiome predicts whether an individual fish has been exposed to the parasite. Our study clarifies how fish, their gut microbiota, and intestinal parasites interact and suggests that the gut microbiome may be an important driver of parasitic success in the gut.

## Methods

### Zebrafish husbandry and *Pseudocapillaria tomentosa* exposure

A total of 210 4-month-old 5D line zebrafish (recipient fish) were randomly divided into six 9-L tanks (35 fish/tank) and were allowed to equilibrate to the tank for 1 week. During the experiment, water conditions were maintained at temperature 27 °C, pH 7.50, and total ammonia 0.19 mg • L^−1^ (measured with pH and ammonia test kits; Aquarium Pharmaceuticals Inc., Chalfont, PA). Fish were fed Gemma Micro 300 (Skretting, Fontaine-lès-Vervins, France) ad libitum twice daily. The total quantity fed daily was ~ 3% fish body weight.

To create an infectious inoculum, we collected feces from ~ 50 5D line zebrafish (donor fish) that had previously been exposed to *P. tomentosa*. These feces were spun at 1500 g for 45 m, and the pellet washed and subjected to an additional spin at 300 g for 10 m to pellet eggs and feces. The eggs and feces mixture was incubated at 27 °C for 7 days to allow the eggs to larvate. Identical methods were used to generate a mock inoculum from ~ 50 5D line fish that had never been exposed to *P. tomentosa*. Before inoculation, the number of larvated eggs was quantified using light microscopy and a volume equivalent to 175 eggs/fish was delivered to tanks. An equivalent amount of unexposed mock inoculum was added to the unexposed tanks. To maximize the potential that fish would become infected, the volume of each tanks was lowered to 4.5 L and the water flow was shut off for 24 h, after which the flow was returned. After exposure, recipient fish were mixed and randomly separated into three 3-L tanks of parasite-exposed (hereafter, exposed) fish (*N* = 105, 35 fish/tank) and three 3-L tanks of parasite-unexposed (hereafter, unexposed) fish (*N* = 105, 35 fish/tank; Additional file 1: Figure S1). Sex ratios were similar between exposed (66 female, 35 male) and unexposed (71 female, 33 male) groups (numbers do not sum to 105 due to mortality, ambiguous sex, and sample removal due to a low number of sequences). The length of fish between exposed and unexposed groups was also similar (mean unexposed 26.39 mm, standard deviation unexposed 1.36, mean exposed 25.97 mm, standard deviation exposed 2.25; Additional file 2).

Fish were progressively evaluated at 0, 7, 10, 21, 30, 43, 59, and 86 days post-initial exposure (dpe). These time points were selected as our prior unpublished work indicated that important histological and microbiologic changes occur at or near these times. Eighteen hours before necropsy, 15 negative control fish (*N* = 5/tank) and 15 *P. tomentosa*-exposed (*N* = 5/tank) fish were isolated and individually housed in 1.8-L tanks for fecal collection. Stool was collected using a new 5-ml sterile transfer pipet from each tank stored at − 20 °C until processing. Nitrile gloves were worn during fecal collection and were changed between exposure groups. With the exception of the pre-exposure time point (day 0), all fish were euthanized by rapid chilling in iced water (2–4 °C) immediately after fecal collection. Fish were subsequently weighed and measured from snout to tail, and their intestines removed for parasitological and histopathological analysis using 70% ethanol-sterilized instruments. Condition factor was calculated using the equation *K* = (weight × 100)/length^3^ as previously described [18]. Day zero animals were returned to their source tank after fecal samples were collected.

To identify variables that may be associated with weight or condition factor, a stepwise regression (R, MASS v7.3.48; [19]) was calculated using the base formulae:

$$ {\displaystyle \begin{array}{l}\mathrm{Weight}={\beta}_0+{\beta}_1\left(\mathrm{sex}\right)+{\beta}_2\left(\mathrm{exposure}\right)+{\beta}_3\left(\mathrm{total}\ \mathrm{burden}\right)+{\beta}_4\left(\mathrm{histopathology}\ \mathrm{score}\right)\ \\ {}+{\beta}_5\left(\mathrm{length}\right)+{\beta}_6\left(\mathrm{sex}:\mathrm{exposure}\right)\end{array}} $$

$$ {\displaystyle \begin{array}{l}\mathrm{Condition}\ \mathrm{factor}={\beta}_0+{\beta}_1\left(\mathrm{sex}\right)+{\beta}_2\left(\mathrm{exposure}\right)+{\beta}_3\left(\mathrm{total}\ \mathrm{burden}\right)+{\beta}_4\left(\mathrm{histopathology}\ \mathrm{score}\right)\\ {}+{\beta}_6\left(\mathrm{sex}:\mathrm{exposure}\right)\end{array}} $$

The stepwise search was performed in both directions, and the final formula generated was used to calculate a linear regression for the response and optimal parameter set (R v3.3.2; [20]). The impact of sex on parasite burden in the exposed fish was evaluated using a Wilcoxon rank sum test in R.

### Microscopic analysis

Following euthanasia, the abdomen of each fish was opened and the entire intestine was removed. Wet mounts were prepared from each intestine by placing the intestine on a glass slide with a 25 × 60 mm coverslip, and the entire intestine was examined with a compound microscope (Leica DMLB, Wetzlar, Germany) at × 200 to quantify the number of total live worms, mature female worms, and dead worms present. After examination, each intestine was preserved in Dietrich’s fixative, processed for histology, and stained with hematoxylin and eosin using our standard protocol [21].

Pathological changes were scored by a pathologist (M.K.) based on examination of tissues from each intestine. Following our previous study [22], two broad categories, inflammation and hyperplastic changes, were scored in zebrafish intestines. A total histopathology score, which is the sum of the inflammation and hyperplasia scores, was also calculated. A description of our scoring criteria follows.

#### Hyperplasia

Normal intestine (score 0)—nuclei are basal, lymphocytes are uncommon within the epithelium, and goblet cells were frequently observed. Mild hyperplasia (score 1)—changes are mild or equivocal, and there is some increase in basal-located nuclei within the mucosal epithelium. Moderate hyperplasia (score 2)—the mucosal epithelium is diffusely thickened, and there are numerous intraepithelial leukocytes composed primarily of lymphocytes and rodlet cells. Severe hyperplasia (score 3)—the layer of basilar nuclei is severely thickened and often extends to near the brush border in some locations, and the mucosal epithelium is markedly thickened at the bases of intestinal folds and bulges into the muscularis. This score (hyperplasia score 3) also includes dysplasia, in which proliferating enterocytes lose their polarity and are poorly organized. Neoplasia (also included in score 3)—small cell carcinomas, with neoplastic enterocytes invading through the lamina propria and tunica muscularis. As only two fish exhibited this change, they were included with the hyperplasia 3 for statistical analyses.

#### Inflammation

While there was an increase in intraepithelial leukocytes within the epithelium of intestines exhibiting hyperplasia, we confine the inflammation category to chronic inflammation of the lamina propria because migration of leukocytes through the epithelium can be a transient, physiological phenomenon. Mild inflammation (score 1)—detectable increase in cellularity of lamina propria due to increased inflammation. Moderate inflammation (score 2)—multifocal regions of increased cellularity of the lamina propria, with localized and clear separation of the epithelial crypts from the tunica muscularis. Severe inflammation (score 3)—similar to 2, except more extensive and diffuse, in which most of the epithelium is clearly separated from the tunica muscularis by inflammation.

### 16S amplicon library preparation and sequencing

Isolation of microbial DNA from fecal samples was performed using the MoBio PowerSoil® DNA isolation kit (MOBIO, Carlsbad, CA, USA) following the manufacturer’s protocol. An additional 10-min incubation at 65 °C before bead beating (0.7-mm garnet beads) was added to facilitate cellular lysis. Immediately following this incubation, the samples underwent bead beating on the highest setting for 20 min using Vortex Genie 2 (Fisher, Hampton, NH, USA) and a 24 sample vortex adaptor (MOBIO). One microliter of DNA was then used as input into triplicate PCR reactions, and the remaining DNA stored at − 20 °C. Amplification of the V4 region of the 16S rRNA was performed as previously described using the 515f and 806r primers [23, 24]. To ensure proper amplification, amplicons were visualized using gel electrophoresis (1% agarose gel) and quantified using the Qubit® HS kit (Life Technologies, Carlsbad, CA, USA) according to the manufacturer’s instructions. A total of 200 ng of amplicon library was pooled and then cleaned using the UltraClean® PCR clean-up kit (MOBIO) and diluted to a concentration of 10 nM. The final pooled and cleaned product was submitted to the Oregon State University Center for Genome Research and Biocomputing (CGRB) for cluster generation and 250-bp paired-end sequencing on an Illumina MiSeq instrument. This generated ~ 9 million 250-bp paired-end sequences from a total of 244 samples. Due to poor quality scores on the reverse reads, only forward reads were input into DADA2 [25] for quality filtering (truncLen = 240, maxEE = 1, truncQ = 11, rm.phix = TRUE), sequence variant calling, chimera filtering (method = “consensus”) and taxonomic assignment against the Silva reference database (v128) [26].

### Microbial community diversity analyses

The sequence variant table generated using DADA2 was imported into R (version 3.3.2) and rarefied to a read depth 5000 counts (using R, vegan v2.4.6) to produce a sequence variant table that contained 4129 sequence variants (Additional file 3). Two zebrafish fecal samples were filtered during rarefaction as they had fewer than 5000 total counts. All downstream analyses were conducted with this rarefied sequence variant table. Alpha-diversity was assessed using Shannon entropy (R, vegan). Stepwise regression was calculated to determine the set of variables that best explained the variance in Shannon entropy. The base formula used for this analysis was as follows:

$$ {\displaystyle \begin{array}{l}\mathrm{Shannon}={\beta}_0+{\beta}_1\left(\mathrm{dpe}\right)+{\beta}_2\left(\mathrm{exposure}\right)+{\beta}_3\left(\mathrm{total}\ \mathrm{burden}\right)+{\beta}_4\left(\mathrm{histopathology}\ \mathrm{score}\right)\\ {}+{\beta}_5\left(\mathrm{condition}\ \mathrm{factor}\right)+{\beta}_6\left(\mathrm{dpe}:\mathrm{exposure}\right)\end{array}} $$

The stepwise search was performed in both directions, and a linear regression was calculated for the best set of predictors.

Bray-Curtis dissimilarity was quantified using vegan and ordinated and visualized using non-metric multidimensional scaling (NMDS). Kendall rank correlations between NMDS dimensions and worm burden and histopathology were calculated using R. To identify host and parasite factors that may contribute to community structure, we used permutational multivariate analysis of variance (PERMANOVA, R vegan) with 5000 permutations. Days post exposure, exposure status, sex, total worm burden, condition factor, total histopathology, and tank, as well as the interaction between days post exposure and exposure status, were used as parameters in this model. The *p* values for the coefficients of this model were corrected using Holm’s method. Beta-dispersion for the exposed and unexposed fish was also calculated using the vegan package with default parameters. An ANOVA was used to determine if there were significant differences in dispersion between groups.

### Generalized linear models and generalized linear mixed effect models

Associations between total parasite burden (burden) and genera abundance (Additional file 4) were quantified using negative binomial generalized linear models (R, MASS v 7.3.48). The negative binomial distribution was deemed appropriate as (1) negative binomial distributions of parasite burden are commonly observed in nature [27], (2) our previous work indicates that *P. tomentosa* burden distributions are appropriately modeled by the negative binomial distribution [28], and (3) parasite burden in the present study was overdispersed (*σ*^2^/*μ* = 9.2; Additional file 5: Figure S2A). A single baseline model was constructed to quantify the association between burden, exposure status, and days post exposure:

1 $$ \mathrm{Burden}={\beta}_0+{\beta}_1\left(\mathrm{exposure}\right)+{\beta}_2\left(\mathrm{dpe}\right)+\varepsilon $$

An additional model was then constructed for each genus that incorporated a genus abundance parameter in addition to exposure status and days post exposure:

2 $$ \mathrm{Burden}={\beta}_0+{\beta}_1\left(\mathrm{genus}\ \mathrm{abundance}\right)+{\beta}_2\left(\mathrm{exposure}\right)+{\beta}_3\left(\mathrm{dpe}\right)+\varepsilon $$

We used the Akaike information criterion (AIC; R, bbmle v 1.0.20) to select the best-fit model for each genus. Genera for which the baseline model was found to be the best fit were discarded. False discovery rate of coefficients (excluding the intercept) was controlled using the qvalue package in R (qvalue v 2.6.0; [29]). Only genera with a prevalence of > 10% were considered for downstream visualization.

To identify genera whose abundances were influenced by parasite burden or histopathological changes that accompany infection, we used zero-inflated negative binomial generalized linear mixed effects models (glmmTMB v 0.2.0 [30]). The glmmTMB package allows the use of two negative binomial distributions in which the variance increases linearly or quadratically with the mean. Rather than selecting one of these profiles, we fit the following model using both approximations of the negative binomial distribution:

$$ \mathrm{Abundance}\sim \mathrm{exposure}+\mathrm{dpe}+\mathrm{burden}+\mathrm{total}\ \mathrm{histopathology}\ \mathrm{score} $$

The intercept of tanks (random effect) was allowed to vary as previous work has indicated that co-housing may influence microbial community composition [31]. For each model, a single zero-inflation parameter was applied to all observations (i.e., ziformula = ~ 1). Fit AIC was calculated as above, and the model that both converged and had the lowest AIC was selected as the best fit. False discovery rate was calculated as above for all *p* values of fixed effects (excluding the intercept). Genera with prevalence in fish > 10% were used in downstream visualization.

### Machine learning to predict exposure, parasite burden, and histopathology

Random forest analysis (R, randomForest v4.6.12; [32]) determined if microbial abundance predicts *P. tomentosa* exposure, burden, or resulting pathology scores. Before building classification trees, the genera abundance table was filtered to exclude genera present in < 10% of fish. A total of 10,000 trees were grown for the classifier, and the default formula ($\sqrt{p}$, where *p* is the number of genera) was used to calculate the number of variables to sample at each split. Sampling was conducted with replacement and a minimum terminal node size of one. During forest construction, variable importance was also calculated (importance = TRUE, nPerm = 1) and the mean decrease in accuracy across all classes was reported. To test the robustness of the classifier to study effects, we predicted the exposure status of a pilot dataset containing 65 *P. tomentosa*-exposed 5D line zebrafish (Additional file 6). Sensitivity was calculated as true positives (true positives/false negatives).

### Phylogenetic assessment of *Mycoplasma* spp.

A phylogeny of *Mycoplasma* spp. 16S rRNA sequences was constructed from all full-length *Mycoplasma* spp. 16S rRNA sequences in SILVA’s All-Species Living Tree Project [26], a *Mycoplasma* sp. sequence described by Burns et al. [22]*,* and experimentally obtained samples from zebrafish in this study. To ensure equitable lengths of 16S sequences, we first trimmed the ARB-aligned SILVA *Mycoplasma* spp. sequences to the V4 in silico using aligned 515F and 806R universal 16S rRNA primer sequences as bounding markers of this region. *Mycoplasma* spp. sequences from SILVA, Burns et al., and our experimental sequences were aligned with NAST aligner using the mothur [33] (v 1.39.3) commands align.seqs (options: flip = TRUE) and filter.seqs (options: vertical = T). The SILVA-aligned *E.coli* was used as the profile alignment for reference. A de novo phylogenetic tree consisting of 122 tips was constructed from the V4 reads using FastTree (v 2.1.10, options –nt –gtr).

## Results

### *P. tomentosa* infection induces progressive weight loss and gut lesions

**Fig. 1:**
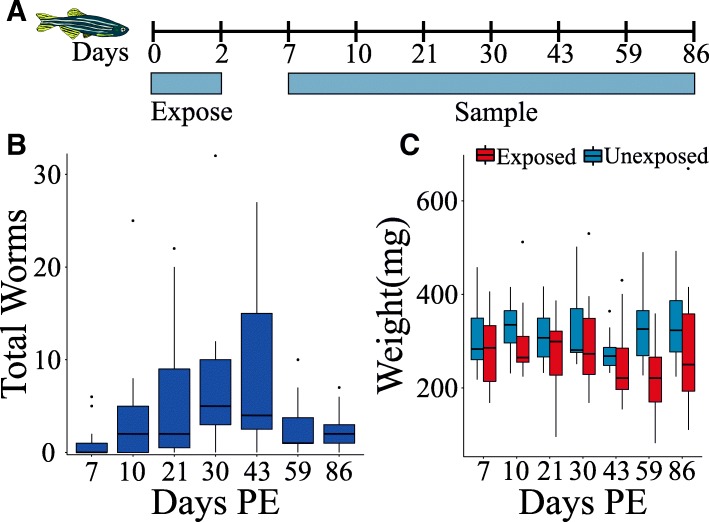
Sampling strategy and physiological manifestations of *P. tomentosa* exposure. **a** Exposure and sampling strategy. **b** The number of worms observed in the intestine of individual fish (wet mount) at each sampling time point. **c** Weights of unexposed (blue boxes) and exposed fish (red boxes) at each sampling time point

To quantify the impact of *P. tomentosa* infection in zebrafish, we followed the progressive impacts of the infection on parasite burden, weight loss, and gut pathology in 105 exposed and 105 unexposed fish. Worm burden was first observed at 7 dpe (Fig. 1b) and peaked at 30 dpe. Early time points were dominated by larval and immature worms (Additional file 5: Figure S2B, Additional file 7: Figure S3A, B), which declined in abundance beginning at 43 dpe. Sexually mature female worms with eggs (Additional file 7: Figure S3C, E) were observed beginning at 30 dpe and were consistently found throughout the remainder of the study (Additional file 5: Figure S2C). Parasite prevalence increased from 47% at 7 dpe to a peak of 93% at 43 dpe before falling slightly to ~ 83% for the remainder of the study (Additional file 5: Figure S2E). Interestingly, despite high prevalence and robust burden, *P. tomentosa* resulted in infrequent mortality (1 of 105) during the experiment. Consistent with our previous work [28], sex did not significantly influence parasite burden (Wilcoxon rank sum, *p* = 0.41; Additional file 5: Figure S2D, F).

Profound weight loss and emaciation is a common sign of severe *P. tomentosa* infection. Exposed fish declined in weight and condition factor, a length normalized measure of weight, as compared to unexposed controls following peak burden (Fig. 1c, Additional file 8: Figure S4). These differences were consistent across male and female fish, indicating that *P. tomentosa* infection effects weight loss in both sexes similarly (Additional file 8: Figure S4). However, this decrease was highly variable across individuals, which indicates that additional factors may impact weight loss in exposed fish. Stepwise multiple linear regression uncovered the set of experimental factors that best predicts and possibly influences fish weight. The optimal regression included the factors sex, histopathology score, and length (mm) (*F*_3, 200_ = 103.3, *p* < 2.2 × 10^−16^, *R*^2^ = 0.60). Sex and histopathology score had significant negative slopes, but fish length had a significant positive slope (Additional file 9: Table S1). These findings indicate that shorter male fish with more severe gut damage tended to weigh less. Similar associations were observed between histopathology, sex, and condition factor (Additional file 10: Table S2).

**Fig. 2:**
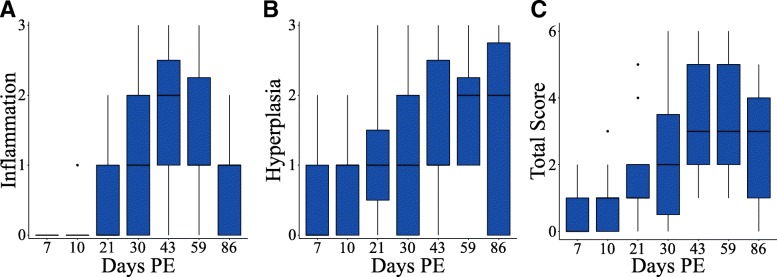
Longitudinal histopathological changes during *P. tomentosa* infection. **a** Inflammation, **b** hyperplasia, and **c** total histopathology score in animals exposed to *P. tomentosa* across the length of experiment

To elucidate the pathological effects of *P. tomentosa* infection on the zebrafish gut, histopathological examination was conducted on intestines collected from zebrafish at each time point. Intestines were scored (0–3) for both inflammation and hyperplasia (Fig. 2a, b). A total histopathology score, which was the sum of the inflammation and hyperplasia scores (range 0–6), was also calculated (Fig. 2c). Throughout the infection, worms were almost exclusively detected in the epithelium and lumen and pathological changes were most often confined to the lamina propria and epithelium. However, two fish exhibited chronic coelomitis (Additional file 7: Figure S3F, G), with a worm in the coelom of one fish, at 43 dpe, and two fish exhibited small cell carcinomas with neoplastic cells invading through the tunica muscularis at 86 dpe.

**Fig. 3:**
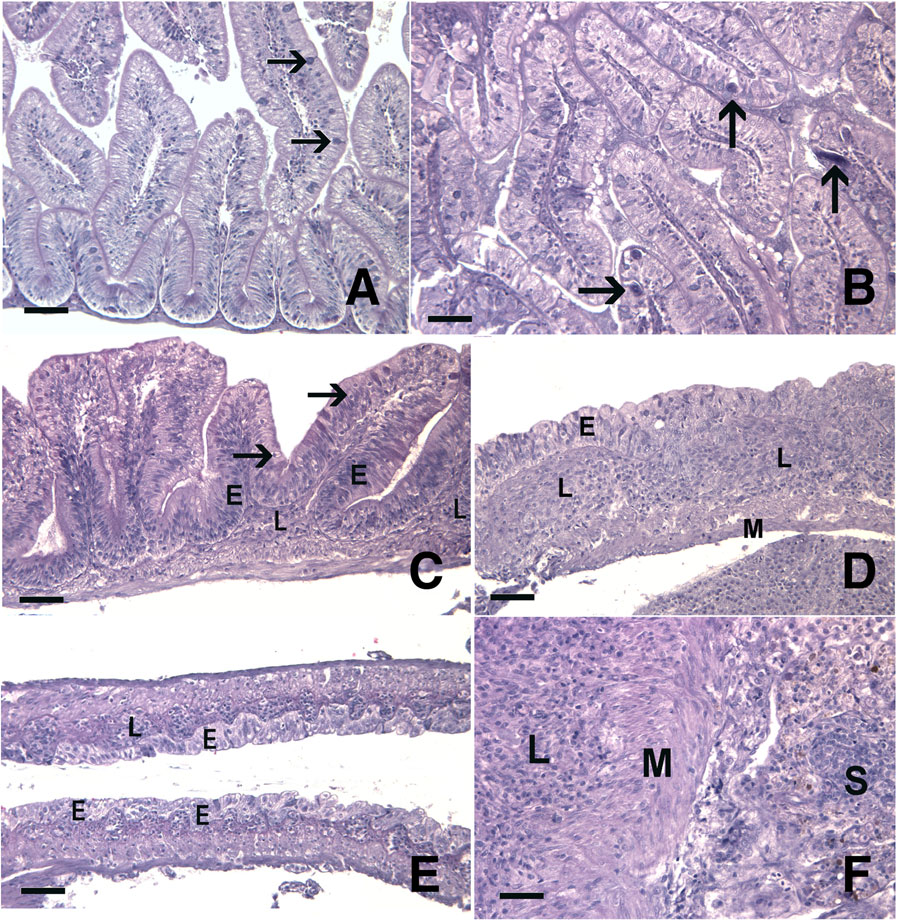
Pathologic changes in fish infected with *Pseudocapillaria tomentosa*. Hematoxylin and eosin stained sections of parasite exposed and unexposed intestines (**a**-**f**). **a** A representative unexposed, control fish with minimal cellularity in the lamina propria and numerous goblet cells (arrows). **b** Intestine of a *P. tomentosa-*exposed fish at 7 dpe exhibiting mild hyperplasia (score 1) and containing numerous larval worms (arrows). **c** An exposed fish at 59 dpe exhibiting severe hyperplasia (score 3) with increased basilar nuclei (E) extending to near the brush border in some locations, numerous rodlet cells (arrows), and expanded lamina propria (L) due to chronic inflammation. **d** Chronic inflammation, inflammation score 3, with extensive expansion of the lamina propria (L), largely dysplastic epithelium (E), and loss of epithelial cell polarity (hyperplasia score 3) in an exposed fish at 59 dpe. **e** Extensive flattening of epithelial folds (E) and moderate expansion of lamina propria (L) due to chronic inflammation (hyperplasia 3, inflammation 2) at 86 dpe. **f** A carcinoma in an exposed fish at 86 dpe with neoplastic epithelial cells proliferating in the lamina propria (L), invading through tunica muscularis (M), and nests of neoplastic cells (S) present in the serosa. Scale bars = 50 μm

Histopathological changes were only observed in exposed animals, and the intestines of all unexposed animals appear clinically normal (Fig. 3a). Early in the infection (e.g., 7 and 10 dpe), pathological changes were observed in many fish. However, the extent of severity at these time points was mild (Fig. 3b), with only two exposed fish scored higher than mild (score 1) for hyperplasia or inflammation. Worms were larger at 21 dpe than at earlier time points, but no sexually mature females were observed. Concurrent with increased worm size and number, 13% of exposed fish were scored with severe (score 3) hyperplasia at 21 dpe. The extent of hyperplasia remained elevated after 21 dpe, with severe hyperplasia manifesting in several individuals at later time points (Fig. 3c). Beginning at 21 dpe, the underlying lamina propria exhibited mild to moderate chronic inflammation (Additional file 7: Figure S3D). The extent of the inflammatory response in the lamina propria increased until 43 dpe. It remained similar at 59 dpe (Fig. 2b, Fig. 3d) but declined at 86 dpe. Despite decreases in worm burden at time points late in infection, total histopathology score remained high largely due to elevated levels of hyperplasia. In addition, some of the most severe lesions were observed at later time points. For example, two fish exhibited prominent flattening of the intestinal folds, one at 43 and one at 86 dpe (Fig. 3e). At the final time point examined (i.e., 86 dpe), intestinal neoplasms were observed in two exposed fish. This diagnosis was confirmed by the observation of de-differentiated epithelial cells invading through the lamina propria and tunica muscularis and into the serosa (Fig. 3f).

### *Pseudocapillaria tomentosa* infection associates with altered microbiome diversity

**Fig. 4:**
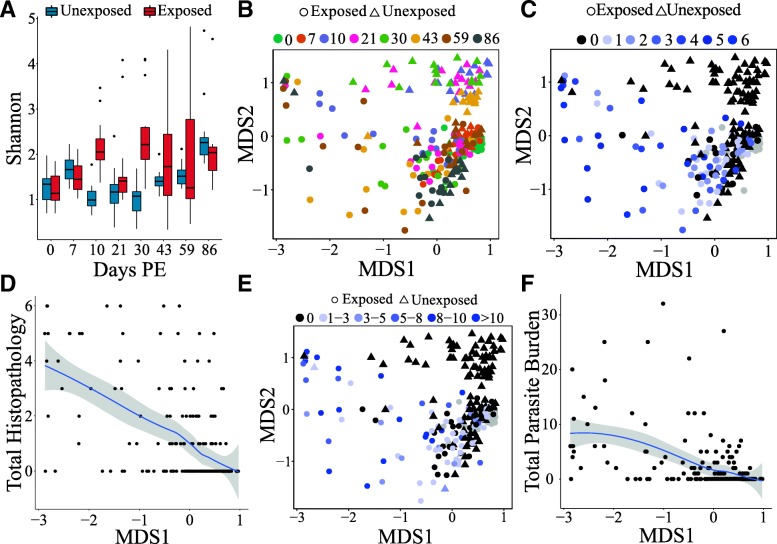
*Pseudocapillaria tomentosa* exposure is associated with altered microbiome composition. **a** Shannon entropy in exposed (red boxes) and unexposed animals. Nonmetric multidimensional scaling plots of exposed and unexposed microbiomes colored by **b** days post exposure, **c** total histopathology score (gray points = N/A), and **e** total parasite burden (gray points = N/A). Correlations between **d** total histopathology score and MDS1 and **f** total parasite burden and MDS1. Blue lines indicate the loess best-fit line and the shaded gray area represents the standard error

To determine if parasite infection or the subsequent histopathological changes in the gut influence microbiome diversity, we calculated Shannon entropy in exposed and unexposed fish. Parasite infection resulted in elevated Shannon entropy at multiple time points when compared to unexposed controls (Fig. 4a, Additional file 11: Figure S5). We used stepwise linear regression to identify parameters that best explained the variation in Shannon entropy. The optimal regression equation included the factors dpe, exposure, parasite burden, and the interaction between time and exposure (*F*_4,201_ = 11.83, *R*^2^ = 0.17, *p* = 1.199 × 10^−08^). Of these parameters, dpe and exposure had significant positive slopes (Additional file 12: Table S3). These results indicated that Shannon entropy was elevated in exposed animals and increased over time upon initiation of infection. The interaction between exposure and time produced a significant negative slope, which indicates that time’s effect on Shannon entropy differs between unexposed and exposed animals.

Exposure to *P. tomentosa* is also associated with altered community structure of the zebrafish gut microbiome (Fig. 4b). Permutational multivariate analysis of variance (PERMANOVA) indicated that microbial communities were significantly stratified by exposure (*F*_1, 108_ = 46.4, *R*^2^ = 0.16, *p* = 1.6 × 10^−03^), days post exposure (*F*_1, 108_ = 15.6, *R*^2^ = 0.05, *p* = 1.6 × 10^−03^), sex (*F*_1,108_ = 3.1, *R*^2^ = 0.01, *p* = 0.03), parasite burden (*F*_1, 108_ = 11.0, *R*^2^ = 0.04, *p* = 1.6 × 10^−03^), condition factor (*F*_1, 108_ = 4.7, *R*^2^ = 0.02, *p* = 5.6 × 10^−03^), and histopathology score (*F*_1, 108_ = 4.8, *R*^2^ = 0.02, *p* = 5.6 × 10^−03^), as well as the interaction between exposure and days post exposure (*F*_1, 108_ = 13.1, *R*^2^ = 0.04, *p* = 1.6 × 10^−03^). Microbial community diversity was not associated with tank (*F*_9, 108_ = 1.2, *R*^2^ = 0.004, *p* = 0.28). Unexposed animals had significantly elevated levels of beta-dispersion with regard to the exposed animals (*F*_1, 235_ = 4.6, *p* = 0.03). We next asked if parasite burden or infection-induced histopathology was associated with the dispersion observed along the primary axis in an MDS analysis (MDS1), as it visually appeared that this axis best stratified the exposed and unexposed animals. Both histopathology (*τ* = 0.41, *p* = 1.7 × 10^−14^; Fig. 4c, d) and total parasite burden (*τ* = 0.38, *p* = 2.9 × 10^−13^; Fig. 4e, f) are significantly associated with MDS1. Together, these data suggest that the microbiome might contribute to *P. tomentosa* success and subsequent infection-associated pathologies.

### The abundance of gut taxa predicts parasite burden

**Fig. 5:**
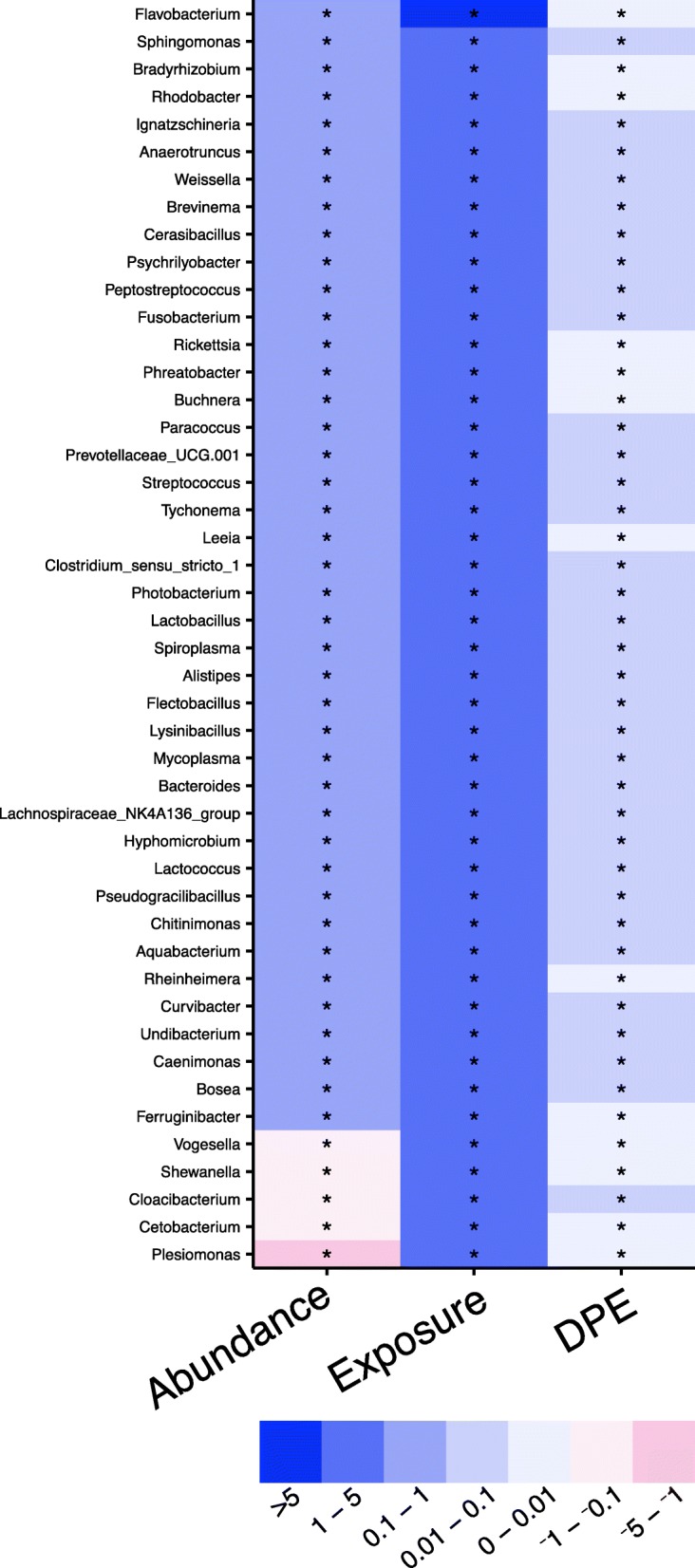
Parasite burden is associated with microbial abundance. A heat map of coefficients from negative binomial generalized linear models with lowest AIC. The color of each cell represents the direction of the slope (red is negative, blue is positive). An asterisk in a cell indicates *q* < 0.15

To elucidate the potential interactions between intestinal parasites and the zebrafish gut microbiome, we quantified how the relative abundance of specific gut taxa covaried with intestinal parasite burden over time, wherein statistical associations between parasite burden and microbiota relative abundance implicate their interaction in the gut. Briefly, a baseline negative binomial generalized linear model with only exposure and days post exposure variables was constructed. A second model that included genus abundance, exposure, and days post exposure was then constructed for each genus individually. The Akaike information criterion was used to select the best-fit model for each genus. We found numerous associations between microbial abundance and parasite burden (Fig. 5, Additional file 13). The significant negative slopes for the abundance of members of the genera *Plesiomonas*, *Shewanella*, and *Cetobacterium* indicate that the abundance of these taxa negatively associates with parasite burden. Conversely, significant positive slopes were observed for the majority (186 of 198) of genera examined, including the abundant genera *Pseudomonas* and *Pelomonas*, which reveal that their abundance increases as parasite burden increases.

Notably, we observed a significant positive slope of abundance for the genus *Mycoplasma*, which previously has been associated with hyperplastic and neoplastic lesions in the intestine of zebrafish [22]. Several of the 16S sequence variants that corresponded to the genus *Mycoplasma* in our study formed a monophyletic clade with the *Mycoplasma* sequence variant previously associated with hyperplastic lesions (i.e., the Burns et al. sequence, Additional file 14: Figure S6). The combined abundance of this clade was also elevated in exposed animals (*W* = 8128, *p* = 0.03; Additional file 15: Figure S7A) and was associated with intestinal hyperplasia score (*τ* = 0.21, *p* = 3.0 × 10^−04^). Similarly, the summed abundance of all the sequence variants corresponding to the genus *Mycoplasma* was also elevated in exposed animals (*W* = 8297, *p* = 0.01; Additional file 15: Figure S7B) and correlated with intestinal hyperplasia score (*τ* = 0.21, *p* = 3.3 × 10^−04^).

**Fig. 6:**
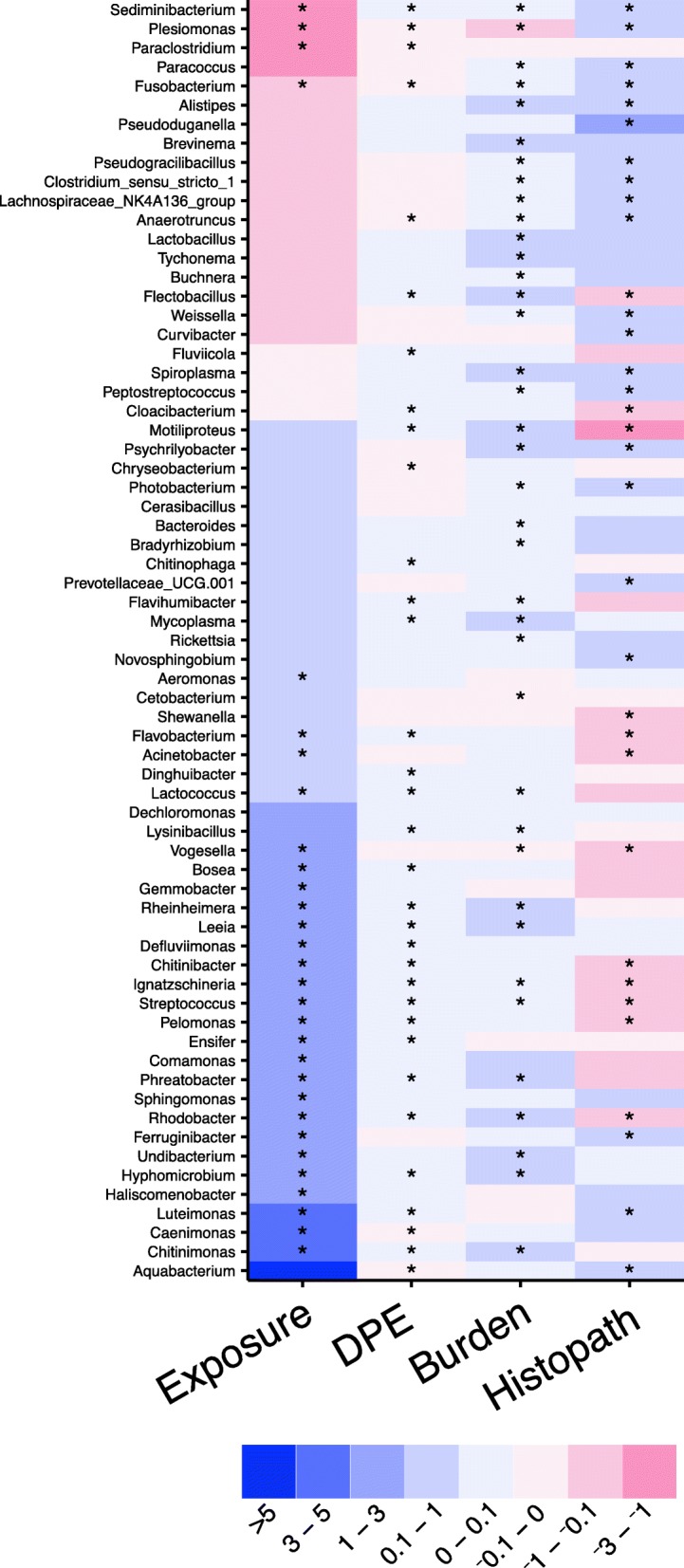
Microbial abundance is associated with parasite burden and histopathology. A heat map of coefficients from converged zero-inflated negative binomial generalized linear mixed effects models with lowest AIC. The color of each cell represents the direction of the slope (red is negative, blue is positive). Gray colored cells indicate that a model without an interaction parameter was selected as it had the lowest AIC. An asterisk in a cell indicates *q* < 0.15

To clarify how changes in the gut microenvironment (i.e., parasite infection) influence microbial communities in the gut, we used negative binomial generalized linear models to quantify the impacts of parasite exposure, burden, histopathology, and time on each genus. The abundance of several taxa is significantly associated with parasite exposure, time, and severity of infection (Fig. 6, Additional file 16). The highly prevalent genus *Plesiomonas* had significantly negative slopes for exposure, time, and parasite burden, indicating that the abundance of this genus is lower in exposed animals and decreases as parasite burden increases. Similarly, the genus *Fusobacterium* also had significant negative slopes for exposure; however, the slope of histopathology score and burden was significantly positive. This finding indicates that while the abundance of these taxa was lower in exposed animals overall, their abundance increases as histopathology score and burden increases. Conversely, the slope of exposure for the genus *Pelomonas* was strongly positive, indicating this taxon may be able to better inhabit the parasitized intestine. However, significant negative slopes were observed for histopathology, suggesting that increasing pathology in the gut during infection associates with decreased abundance of this taxon. Several other taxa, including the highly abundant genus *Aeromonas*, exhibited similar patterns of association with exposure, which indicates these genera are also increased in exposed animals. Together these data showed that *P. tomentosa* exposure associates with altered abundance of specific microbial taxa, and that this may be due, in part, to histopathological changes in the gut that occur during infection.

### Microbiome composition distinguishes parasite exposure

Given the associations between microbial abundance and parasite burden we observed above, we next asked if microbial abundance as measured in stool predicts an individual’s exposure to *P. tomentosa* using machine learning with random forest analysis. Briefly, a random forest classifier was built for exposure status using genera with a prevalence > 10% as predictors. The resulting classifier accurately predicted exposure status with an out-of-bag error rate of 10.1%. Consistent with the results of the regression analysis, the genera *Plesiomonas*, *Pseudomonas*, *Pelomonas*, *Ensifer*, and *Undibacterium* were the variables with highest importance (i.e., highest mean decrease in accuracy; Additional file 17: Figure S8) indicating that removal of these taxa for our classifier resulted in the greatest decrease in the accuracy of classification.

While the random forest classifier performed well on our data set, we questioned whether the information in the classifier was pertinent for diagnosing exposure status in new data. To obtain insight into the robustness of the classifier, we predicted exposure status of zebrafish from an independent, unpublished study. All individuals in this study were exposed to and infected with *P. tomentosa*; thus, if our classifier performed similarly on this dataset, we would expect it to predict ~ 59 of these fish had been exposed to the parasite (i.e., 10% error). Our classifier predicted that 63 of the 65 fish from this cohort were exposed to the pathogen, which corresponds to a sensitivity of ~ 97%. Collectively, these results suggest that passive microbiome sampling (i.e., collecting fecal samples from zebrafish tanks) may robustly and accurately predict exposure to *P. tomentosa* without the need for destructive techniques.

## Discussion

A growing body of evidence suggests that the gastrointestinal microbiome mediates parasitic infections and their impact on host physiology [14, 34–36]. Microbiota that interacts with intestinal helminths represents putative resources for the treatment, prevention, and diagnosis of parasitic infections. Such resources are especially important to identify given the global and rapid rise of anthelmintic drug resistance [6, 37]. Yet, relatively little investigation of the gut microbiome-parasite dynamic has been conducted [38–41]. This study represents, to our knowledge, the most extensive longitudinal assessment of host-parasite-microbiome dynamics to date. By applying robust regression approaches, we identified associations between parasite burden, infection associated pathologies, and host gut microbiome composition, which suggests that microbiome taxa might inhibit or enhance parasite success in the gut. Finally, we used machine learning to develop a diagnostic algorithm, based on our novel non-destructive sampling methodology that is able to predict *P. tomentosa* exposure status in zebrafish.

We find that the infection results in restructuring of the gut microbiome and that the gut microbiome diversifies in association with parasitic burden and severity of histopathology. Analogous studies in mammals found similar, though inconsistent, changes in the gut microbiome upon infection by helminths [14], wherein infection associates with increases in alpha-diversity in some studies [10] and decreases in others [11, 42]. Additionally, specific alterations in microbial taxonomic abundance after experimental infection with *Trichuris suis* [35], *T. muris* [42], and *Heligmosomoides bakeri* [43] have been reported. Unlike prior work, our study design enabled consideration of how the microbiome varies over the course of parasitic infection and included comparisons with histopathological changes. In zebrafish, exposure is associated with increased microbial alpha-diversity and altered beta-diversity, which indicates that infection with *P. tomentosa* results in a restructuring of the gut microbiome. Increased alpha-diversity has been associated with intestinal helminth infection and has been hypothesized to be due to altered gut immune environment in infected individuals [10, 39]. Concordantly, prominent inflammation and hyperplasia in the gut of exposed fish corresponded with maximal alpha-diversity. We cannot, however, rule out possible confounding factors including the contribution of the parasite’s microbiome or the inoculum’s microbiome in our experiments. We also observed significant elevation of beta-dispersion in unexposed fish and associations between days post exposure and microbiome beta-diversity. While the cause of this variation is unclear, it is notable that there are two distinct clusters of unexposed fish (cluster 1 comprising days 10, 21, 30, 43 dpe and cluster 2 comprising days 0, 7, 59, 86 dpe) raising the possibility that these changes represent enterotype switching [44, 45]. However, we cannot exclude other possible contributors to this variation such as aging [46], random drift, or unmeasured changes in the fish’s external environment. Moreover, as we focus primarily on associations between the microbiome and parasite burden and intestinal pathology, it is unlikely that this variation would significantly impact the interpretation of our results.

We also observed variation in parasite burden and the extent of intestinal pathology scores across individuals throughout infection. While several factors could contribute to this variation, we consider that the composition of the gut microbiome may be important in determining the colonization efficiency and pathogenicity of helminth parasites. In *Drosophilia neotestacea*, a maternally transmitted bacterium protects the fly from the helminth parasite *Howardula aoronymphium* [36]. Similarly, *Lactobacillus casei* and *Bifidobacterium animalis* reduce *Trichuris spiralis* [47] and *Strongyloides venezuelensis* [48] burden in mice, respectively. Relatedly, *T. muris* requires the intestinal microbiome to establish infections in mice [34] and germ-free mice infected with *H. bakeri* harbor fewer worms than conventionally raised animals [49]. Using a regression modeling approach, we identified both positive and negative correlations between worm burden and the abundance of specific genera. These results suggest that interactions that either promote or prohibit infection may exist between microbiota and parasitic infection or development in the zebrafish gut. These observations may extend from several possibilities: (1) specific taxa promote or disrupt parasite colonization, growth and development [34, 47, 48], (2) some taxa may be better adapted to the altered gut microenvironment during infection (e.g., inflammation) and concordantly increase in abundance [50], (3) the parasite either opens niche space for microbial taxa to differentially colonize or destroys niche space of resident bacteria, or (4) the composition of the parasite’s own microbiome varies over the course of infection. Changes in appetite or frequent coprophagy in exposed fish may also influence some of the patterns observed here. Further investigation that determines if these correlations reflect causal relationships between microbial abundance in the gut microbiome and *P. tomentosa* infectivity or fecundity is important because, if causative, these associations would represent therapeutic targets for the treatment or prevention of helminth infection.

Throughout this experiment, we observed increasing hyperplasia in the gut epithelium and, at the terminal time point, identified neoplasms. These observations are consistent with a previous work that found infected zebrafish infected with *P. tomentosa* and exposed to a low dose of the carcinogen dimethylbenze[a]antracene had higher incidence of gastrointestinal tumors than similarly exposed fish that were not infected [17]. However, in conducting a large retrospective study of cases of neoplasms from the Zebrafish International Resource Center (https://zebrafish.org/), we found that neoplasms occurred in many laboratories with no history of *P. tomentosa* infections [51]. These results indicate there likely exist other causes of zebrafish intestinal neoplasms. An emerging hypothesis posits that these lesions are caused by *Mycoplasma* spp. [22]*. Mycoplasma* spp. associate with genetic instability and oncogenesis in cell culture [52] and neoplastic lesions in zebrafish [22]. In our study, the genus *Mycoplasma* was increased in *P. tomentosa*-exposed fish and correlated with hyperplasia. Importantly, the *Mycoplasma* spp. sequence variants from our study were closely related to those associated with neoplasms [22]. We observed aggressive, unequivocal tumors at 3 months after exposure to *P. tomentosa*. While prevalence of neoplasms was low in our study (2 of 30 fish examined at 86 dpe), this was similar to the prevalence reported by Burns et al. at 8–10 months after *Mycoplasma* spp. exposure. Given the association between *P. tomentosa* infection and neoplasms [17], we speculate that *Mycoplasma* spp. may be the underlying initiator of these tumors and that the worm, through its associated proliferative changes, acts as a potent promoter. This may be the case with other helminths that are associated with cancers in humans [53]. However, while we did find associations between hyperplasia and *Mycoplasma* spp. abundance, these correlations were relatively modest; thus, we cannot rule out the contribution of other bacteria such as *Pseudomonas* spp. [54] or host innate immune responses [55] in the development of these neoplasms. Regardless of the specific causative agent, this study highlights the potential of zebrafish as a model for spontaneous bacterially induced tumorigenesis in the gut.

One of the goals of clinical microbiome research is to develop microbiome-based diagnostics of disease. Current diagnostic procedures for *P. tomentosa* involve euthanizing several individuals from a zebrafish colony and visually examining the intestine for evidence of parasite infection. Although effective, non-destructive diagnostics are considered preferable as they reduce animal use. Machine learning has previously been used to classify disease risk based on the microbiome [56]. Following this work, we built a random forest classifier that identified fish exposed to *P. tomentosa* with high accuracy. Importantly, this classifier performed equally well on a small-unpublished dataset indicating that it is robust to study effects. However, given the variability of zebrafish gut microbiomes across facility and strain [57], data from additional zebrafish facilities and strains may be needed to optimize the accuracy of this classifier. In addition, it is unclear if the parameters that influence the model are uniquely diagnostic of *P. tomentosa* or rather diagnostic of a disturbed gut microenvironment. For example, the genera *Plesiomonas* and *Pseudomonas* were both highly important variables in our classifier. In this study, the abundance of *Plesiomonas* was decreased and the abundance of *Pseudomonas* was increased in exposed animals relative to unexposed controls. We have previously observed increased *Pseudomonas* and decreased *Plesiomonas* in antimicrobial exposed fish [58] suggesting that this pattern may be an indicator of disturbed guts rather than parasite infection specifically. A more general classifier of disturbed guts may, in fact, be more useful as this could help identify specific tanks of zebrafish that are unlikely to be suitable for experimentation. Future work should determine the specificity of this classifier in the context of multiple diverse exposures.

It is also critical that future work strives to determine if the perturbation of the gut microbiome by *P. tomentosa* affects zebrafish physiology and other health parameters. *P. tomentosa* is a frequent parasite found in zebrafish research facilities, and thus challenges the maintenance of functional experimental animal colonies. In addition to directly endangering animal health, *P. tomentosa* infections may introduce potential confounding experimental results, especially in the case where the infection is cryptic [15]. Given that disruption of the zebrafish gut microbiome is linked to altered host physiology [59–62], behavior [63], and disease [64, 65], it is possible that the effect *of P. tomentosa* infection on host physiology is driven in part due to changes in the microbiome. We found that infection resulted in restructuring of the microbiome that persisted over the duration of the experiment, even as burden decreased during later time points. However, because infection was never cleared in these fish, the resilience of the microbiome upon resolution of parasite infection either naturally or through treatment [66] remains unclear. If the microbiome remains permanently altered following infection, these fish may be unsuitable for subsequent investigations.

The use of zebrafish as a model of host-microbiome interactions is increasing. However, the zebrafish gut microbiome is taxonomically distinct from that of mammals [57, 67–69]. Despite these taxonomic differences, substantial evidence exists indicating that mammal and zebrafish microbiomes share functional similarities and conserved responses to perturbation. For example, zebrafish were used to demonstrate that a bacterial protein found in the guts of fish induces the expansion of pancreatic β cells during development [70]. Although the zebrafish taxa that encode these genes are not prevalent in the human gut microbiome, homologs of this protein that are expected to perform similar functions are found in human microbiomes. The microbiomes of zebrafish and humans also respond similarly to various stimuli including increasing levels of dietary fat [71, 72] and toxicant exposure [58, 73]. In addition, mammalian microbes can colonize the zebrafish gut and vice versa [74, 75] and these microbes elicit similar immune responses in both fish and mammals [76]. Collectively, these studies indicate that while the precise taxonomic changes may vary between fish, humans, and other mammals, the functional outcomes may remain largely consistent. Thus, while exact taxonomic associations observed in this study may not manifest in mammalian systems, there may exist analogous interactions that do. Moreover, if any of the associations observed in this study are driven by microbial production of compounds that inhibit helminth growth, then we may be able to use these compounds to treat helminth infection across a broad range of hosts. Concordantly, several broad-spectrum anthelmintic compounds have been isolated from host-associated and free-living microbes [77–80]. Ongoing work in our group aims to uncover the molecular mechanisms underpinning the associations described herein with the ultimate goal of identifying novel broad-spectrum anthelmintic compounds that can promote human and animal health.

## Conclusions

Collectively, these experiments demonstrate that infection with *P. tomentosa* alters the zebrafish gut microbiome and uncovered microbial taxa that might influence parasite success and virulence in the gut. Our work also establishes the utility of the microbiome as a potential diagnostic for *P. tomentosa* exposure. Although it is unlikely that any specific results (i.e., altered taxa) can be broadly generalized to other intestinal helminth infections, we may be able to use zebrafish to answer fundamental questions about how parasites, associated pathological changes, hosts, and the gut microbiome interact. Future work will clarify the directionality of associations between the microbiome, parasite success, and pathology.

## Additional files

Additional file 1: **Figure S1.** Parasite exposure. A diagram of the methodology used to expose fish to parasite or mock inoculum. (PDF 10870 kb)

Additional file 2: **Table S4.** Microbiome sample metadata table (XLSX 54 kb)

Additional file 3: Tab delimited rarefied sequence variant abundance table (TXT 1959 kb)

Additional file 4: Tab delimited genus abundance table (TXT 282 kb)

Additional file 5: **Figure S2.** Parasite burden across time. A) A histogram of total worm burden in exposed fish. B) Total immature worm burden by wet mount C) Total mature worm burden by wet mount. D) Total worm burden by wet mount in male and female fish. E) Parasite prevalence in exposed animals by day. F) Total worm count by histology in male and female zebrafish. (PDF 63 kb)

Additional file 6: Supplemental text. (DOCX 149 kb)

Additional file 7: **Figure S3.** Microscopic examination of *P. tomentosa* infected intestines. Wet mounts (A-C) of and histological sections (D-G) of *P. tomentosa* infected zebrafish intestines. A) Larval worms (arrows) in intestine at 7 dpe. B) Immature worms at 21 dpe (bar = 100 μm). C) Adult female and free eggs (arrows) at 43 dpe (bar = 100 μm). D) Structures consistent with apoptotic bodies (A) and rodlet cells (arrow) observed in epithelium. E) Sexually mature female worms and free eggs (arrows) in intestine at 43 dpe (bar = 25 μm). F) Coelomitis (arrows), with chronic inflammation in the serosal lining and G) worms in a fish at 43 dpe. Scale bars = 50 μm unless otherwise indicated. (PDF 28169 kb)

Additional file 8: **Figure S4.** Parasite exposure associated with decrease in weight and condition factor if zebrafish. A) Weight of *P. tomentosa* exposed (red boxes) and unexposed (blue boxes) zebrafish. B) Condition factor of *P. tomentosa* exposed and unexposed zebrafish. Plots are split to demonstrate the impact of exposure on female (F) and male (M) fish. (PDF 46 kb)

Additional file 9: **Table S1.** Coefficients table for weight linear regression (XLSX 36 kb)

Additional file 10: **Table S2.** Coefficients table for condition factor linear regression (XLSX 32 kb)

Additional file 11: **Figure S5.** Temporal variation in microbiome diversity in exposed and unexposed fish. Nonmetric multidimensional scaling plots of A) unexposed and B) exposed zebrafish gut microbiomes colored by days post exposure. (PDF 42 kb)

Additional file 12: **Table S3.** Coefficients table for Shannon entropy linear regression (XLSX 35 kb)

Additional file 13: Coefficients table for burden generalized linear models (TXT 56 kb)

Additional file 14: **Figure S6.** *Mycoplasma* spp. sequence variants are closely related to sequence variants associated with gut tumors formation in zebrafish. A phylogenetic tree of sequence variants associated with the genus *Mycoplasma* and *Mycoplasma* spp. 16S rRNA gene sequences from the Silva database. Blue colored branches and labels indicate sequence variants from the current study, and red branches and tip labels are from a *Mycoplasma* sp. associated with tumorigenesis in zebrafish. (PDF 31 kb)

Additional file 15: **Figure S7.** *Mycoplasma* sequence variant abundance is elevated in exposed animals. Boxplots of A) *Mycoplasma* spp. Burns et al. clade members and B) the combined abundance of all *Mycoplasma* sequence variants. Red dots represent the abundance in animals that presented with neoplasms. (PDF 71 kb)

Additional file 16: Coefficients table for genus zero-inflated generalized linear models (TXT 81 kb)

Additional file 17: **Figure S8.** Random forest variable importance. A variable importance plot for the top 15 most important genera. Mean decrease in accuracy is scaled by variable standard deviation. (PDF 32 kb)
