# Supplementary material for: A longitudinal assessment of host-microbe-parasite interactions resolves the zebrafish gut microbiome’s link to Pseudocapillaria tomentosa infection and pathology
Source: Microbiome. 2019 Jan 24;7:10. doi: 10.1186/s40168-019-0622-9 (PMC6346533; doi:10.1186/s40168-019-0622-9)
Supplement: Supplementary file 1 — Figure S1. Parasite exposure. A diagram of the methodology used to expose fish to parasite or mock inoculum. (PDF 10870 kb) [file 40168_2019_622_MOESM1_ESM.pdf]

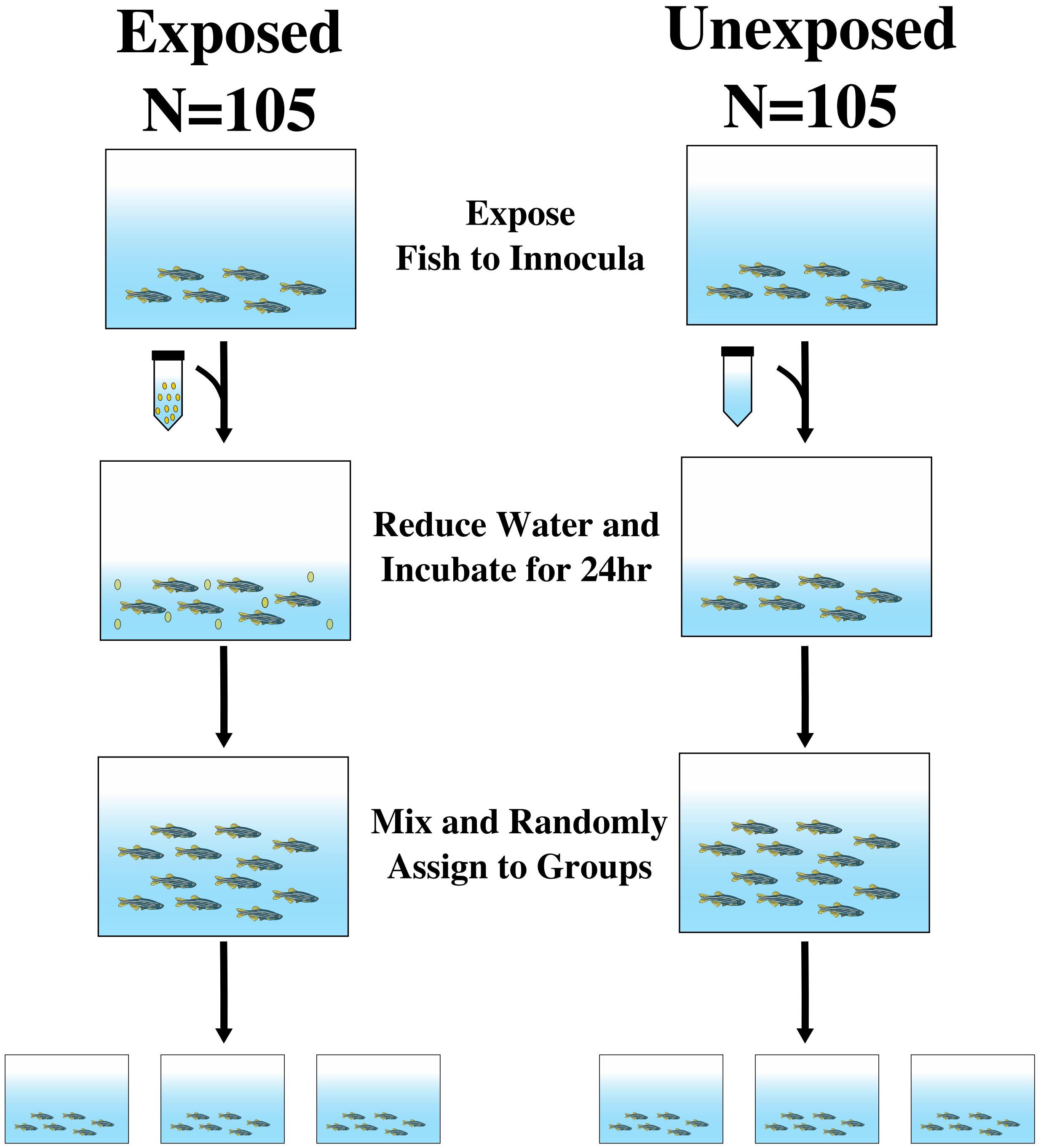

Supplemental Figure 1
