# Supplementary figures and images for: A longitudinal assessment of host-microbe-parasite interactions resolves the zebrafish gut microbiome’s link to Pseudocapillaria tomentosa infection and pathology
Source: Microbiome. 2019 Jan 24;7:10. doi: 10.1186/s40168-019-0622-9 (PMC6346533; doi:10.1186/s40168-019-0622-9)

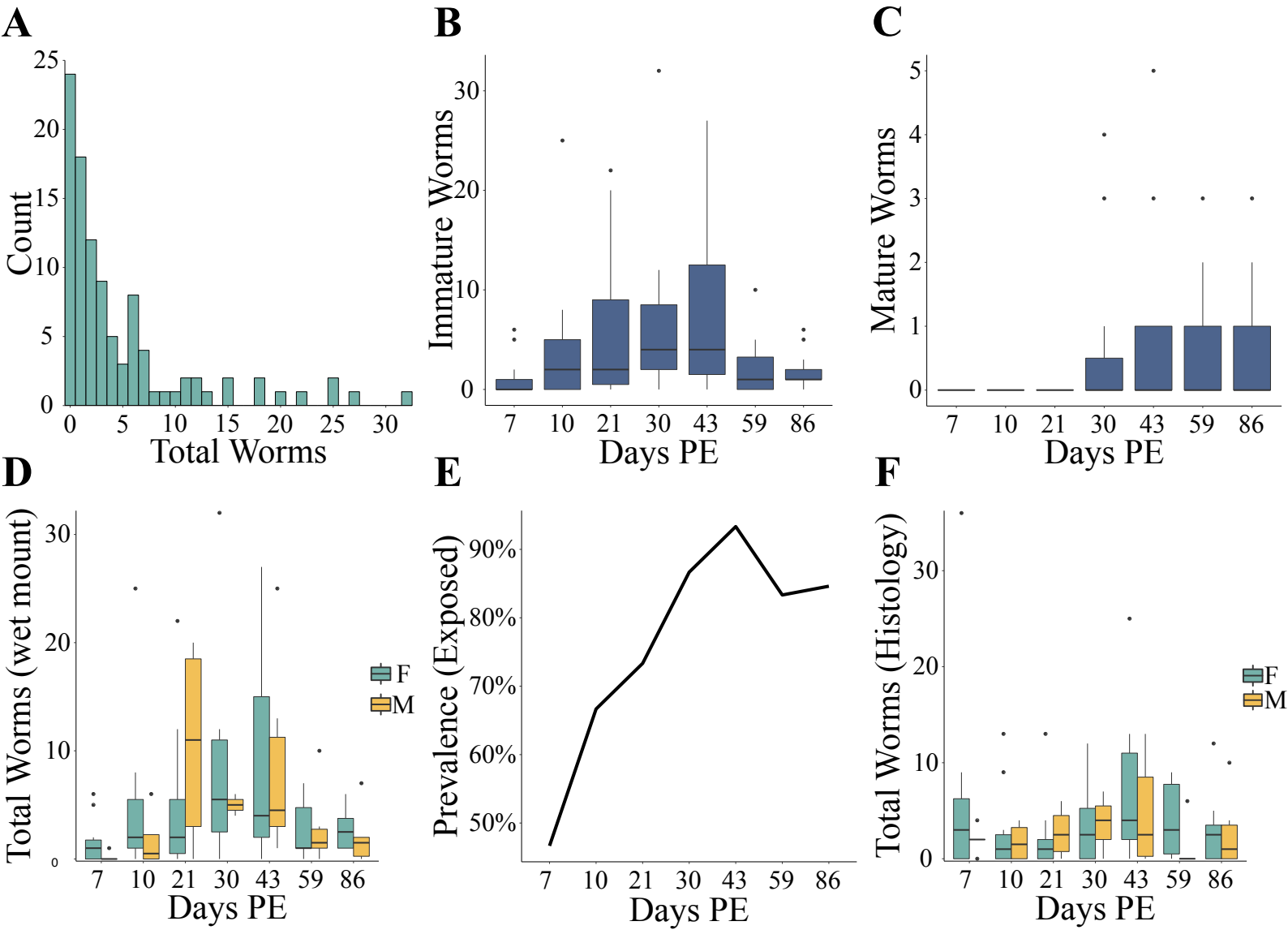

**Supplemental Figure 2**

Supplement: Supplementary file 5 — Figure S2. Parasite burden across time. A) A histogram of total worm burden in exposed fish. B) Total immature worm burden by wet mount C) Total mature worm burden by wet mount. D) Total worm burden by wet mount in male and female fish. E) Parasite prevalence in exposed animals by day. F) Total worm count by histology in male and female zebrafish. (PDF 63 kb) [file 40168_2019_622_MOESM5_ESM.pdf]

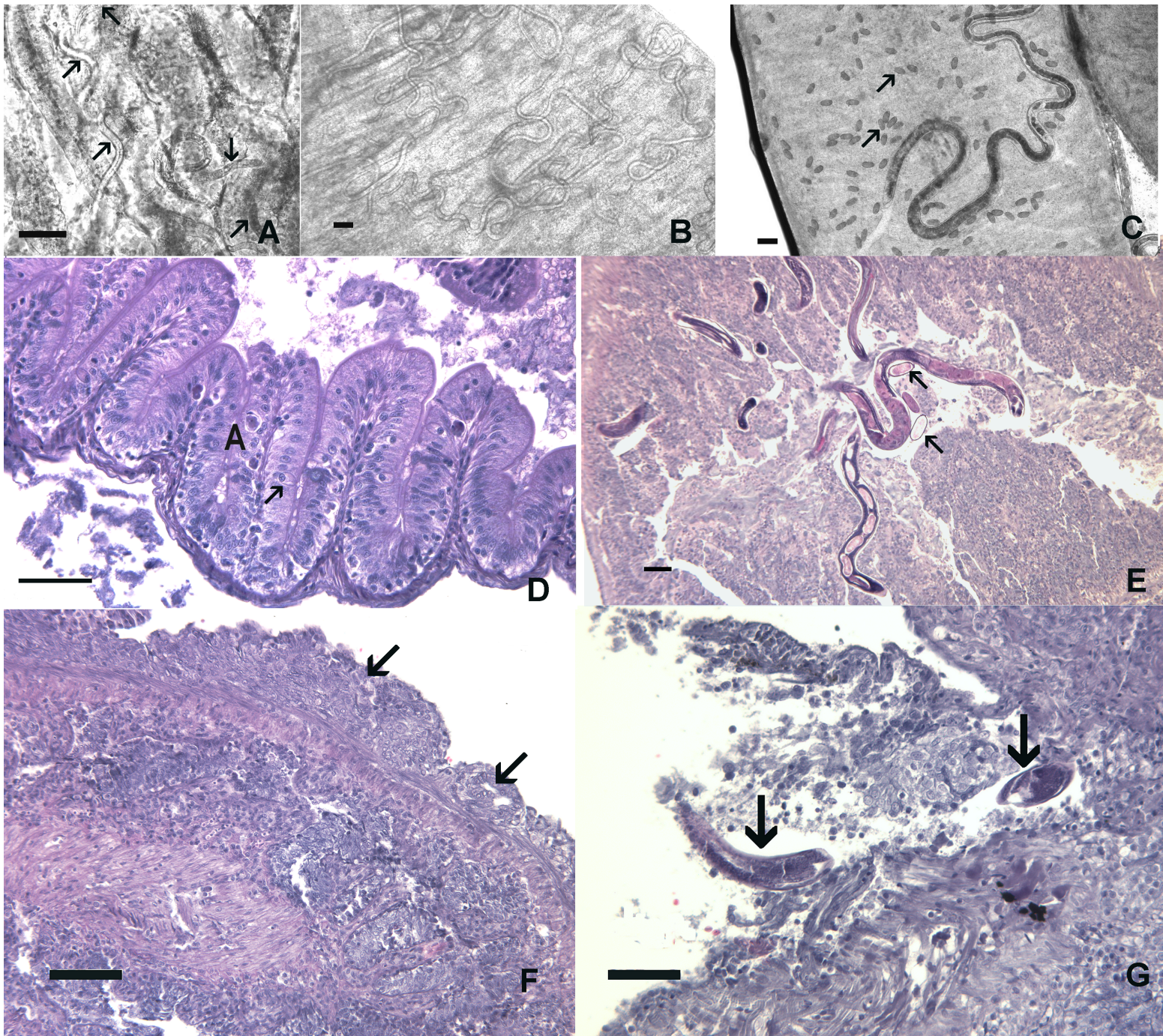

**Supplemental Figure 3**

Supplement: Supplementary file 7 — Figure S3. Microscopic examination of P. tomentosa infected intestines. Wet mounts (A-C) of and histological sections (D-G) of P. tomentosa infected zebrafish intestines. A) Larval worms (arrows) in intestine at 7 dpe. B) Immature worms at 21 dpe (bar = 100 μm). C) Adult female and free eggs (arrows) at 43 dpe (bar = 100 μm). D) Structures consistent with apoptotic bodies (A) and rodlet cells (arrow) observed in epithelium. E) Sexually mature female worms and free eggs (arrows) in intestine at 43 dpe (bar = 25 μm). F) Coelomitis (arrows), with chronic inflammation in the serosal lining and G) worms in a fish at 43 dpe. Scale bars = 50 μm unless otherwise indicated. (PDF 28169 kb) [file 40168_2019_622_MOESM7_ESM.pdf]

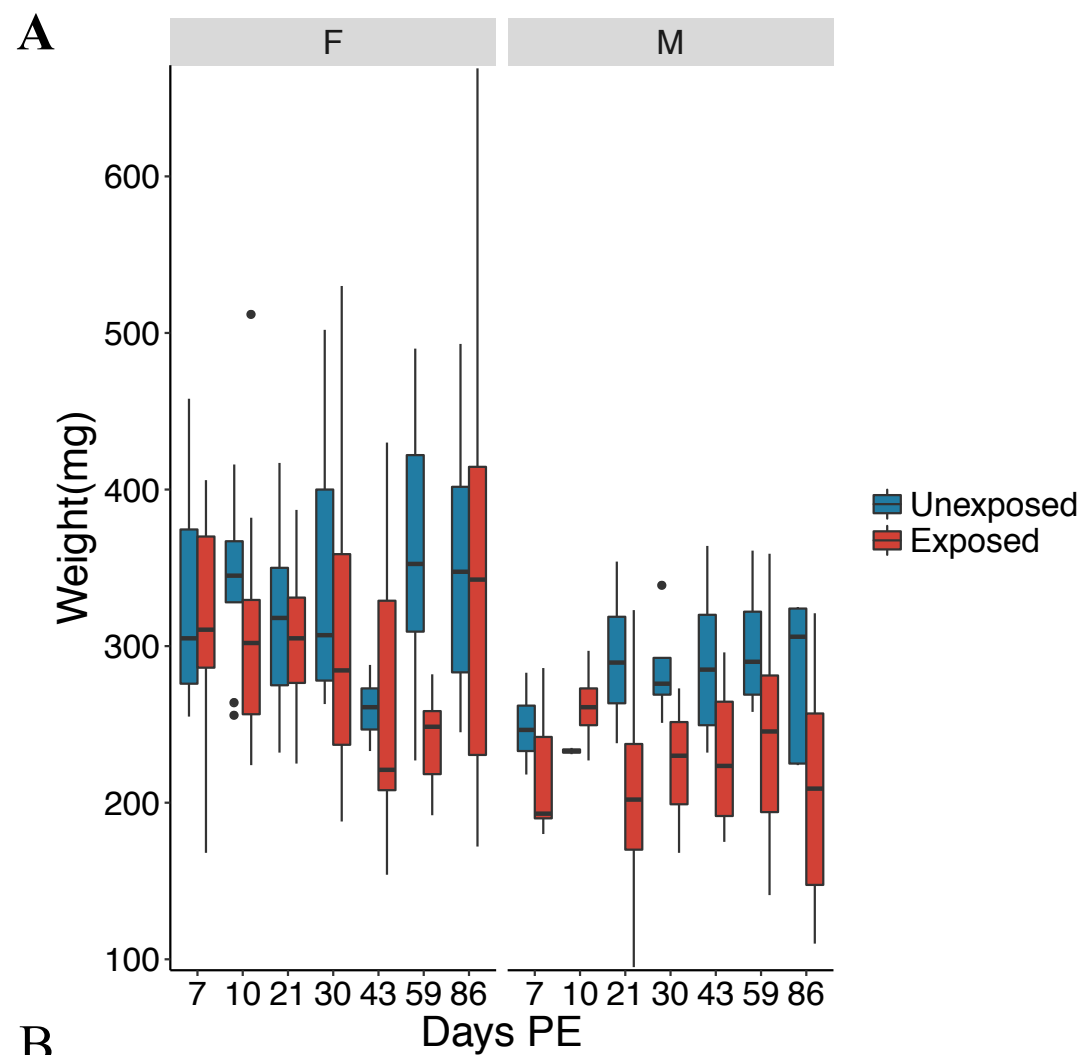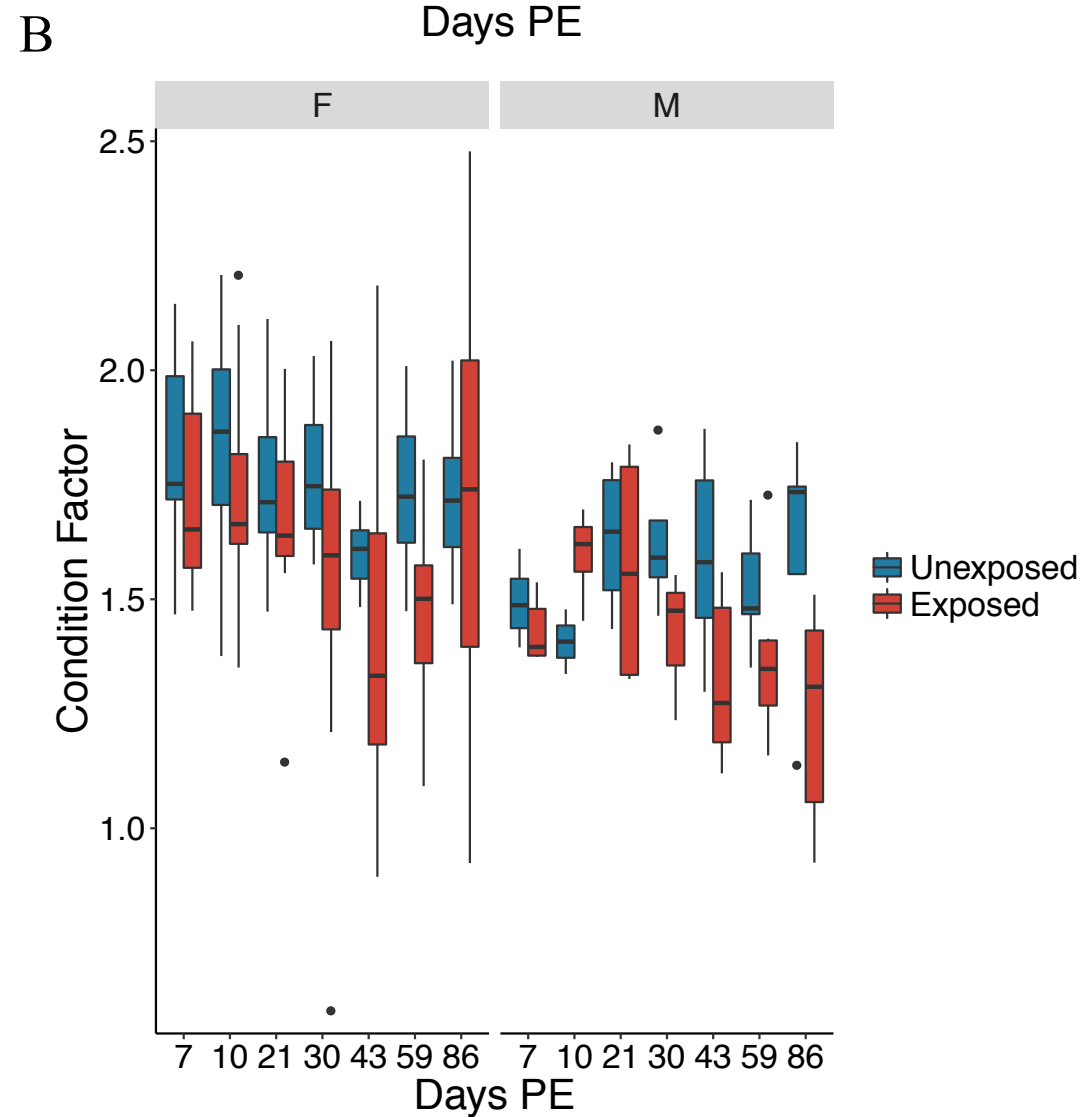

**Supplemental Figure 4**

Supplement: Supplementary file 8 — Figure S4. Parasite exposure associated with decrease in weight and condition factor if zebrafish. A) Weight of P. tomentosa exposed (red boxes) and unexposed (blue boxes) zebrafish. B) Condition factor of P. tomentosa exposed and unexposed zebrafish. Plots are split to demonstrate the impact of exposure on female (F) and male (M) fish. (PDF 46 kb) [file 40168_2019_622_MOESM8_ESM.pdf]

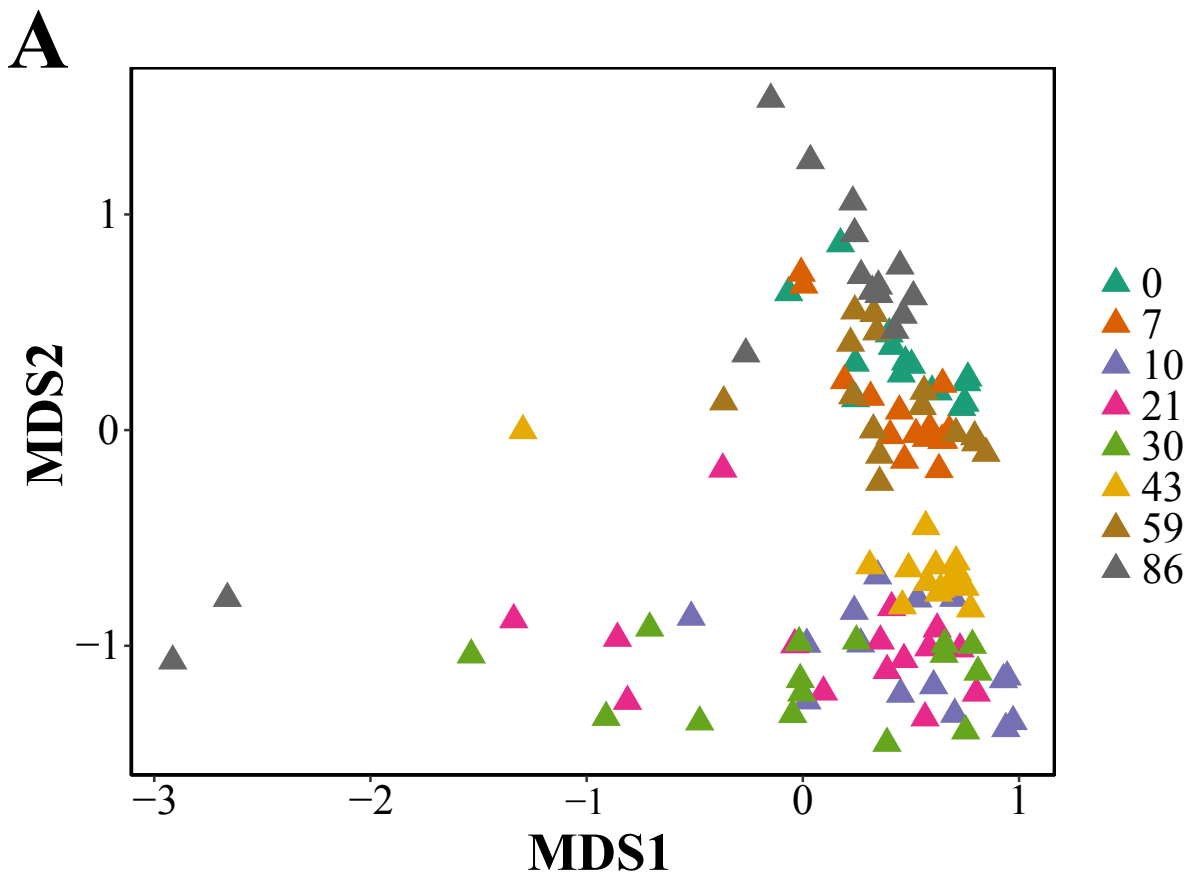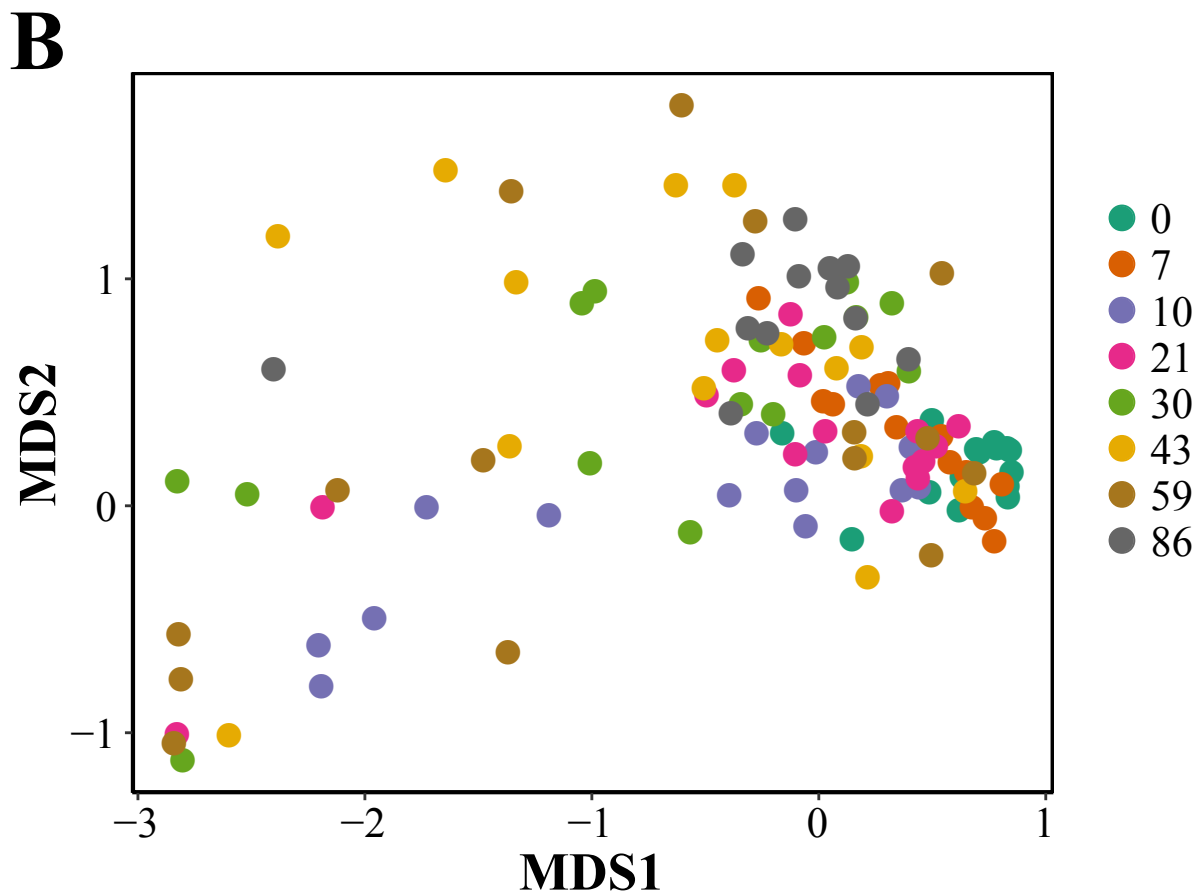

**Supplemental Figure 5**

Supplement: Supplementary file 11 — Figure S5. Temporal variation in microbiome diversity in exposed and unexposed fish. Nonmetric multidimensional scaling plots of A) unexposed and B) exposed zebrafish gut microbiomes colored by days post exposure. (PDF 42 kb) [file 40168_2019_622_MOESM11_ESM.pdf]

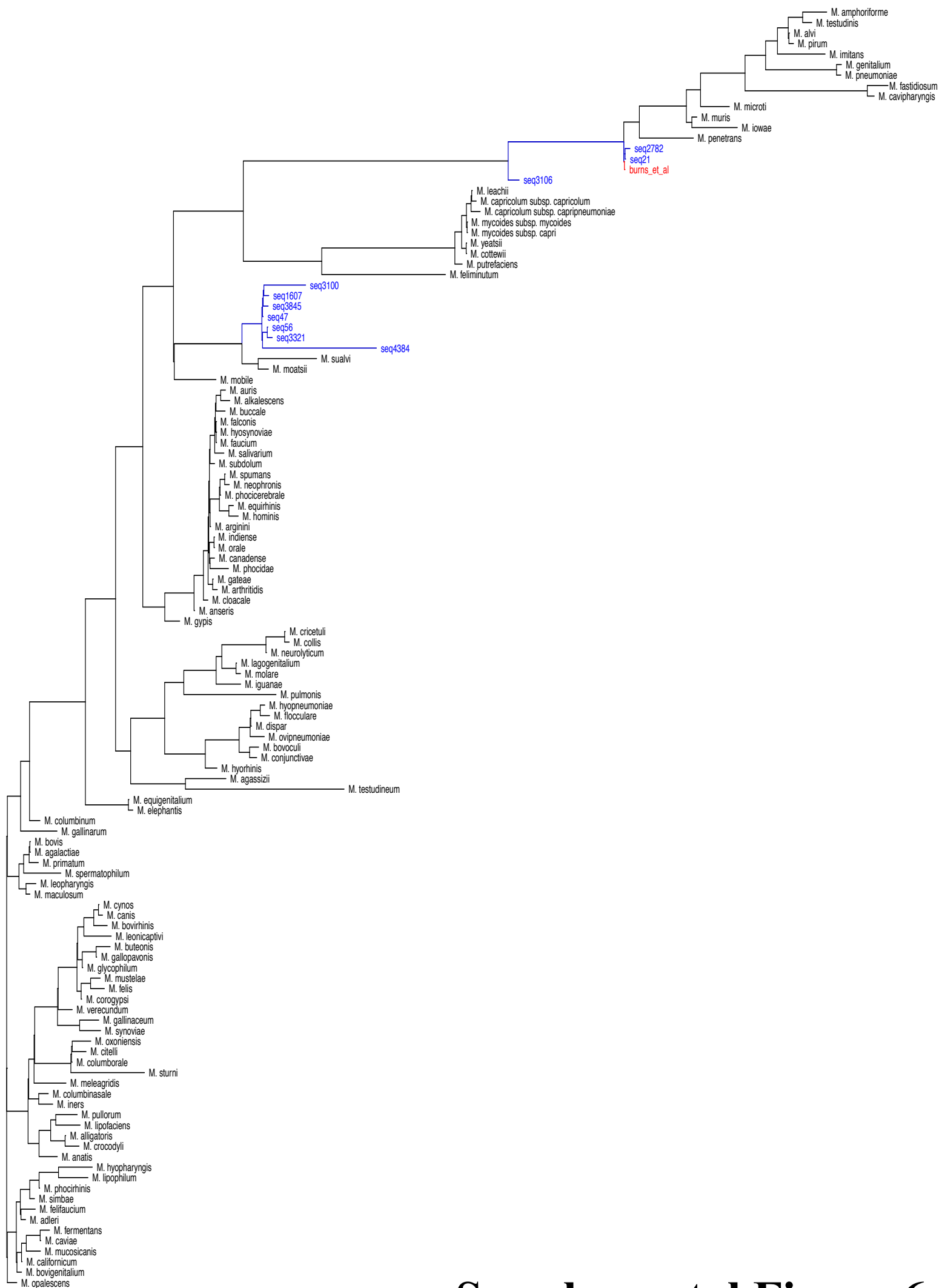

**Supplemental Figure 6**

Supplement: Supplementary file 14 — Figure S6. Mycoplasma spp. sequence variants are closely related to sequence variants associated with gut tumors formation in zebrafish. A phylogenetic tree of sequence variants associated with the genus Mycoplasma and Mycoplasma spp. 16S rRNA gene sequences from the Silva database. Blue colored branches and labels indicate sequence variants from the current study, and red branches and tip labels are from a Mycoplasma sp. associated with tumorigenesis in zebrafish. (PDF 31 kb) [file 40168_2019_622_MOESM14_ESM.pdf]

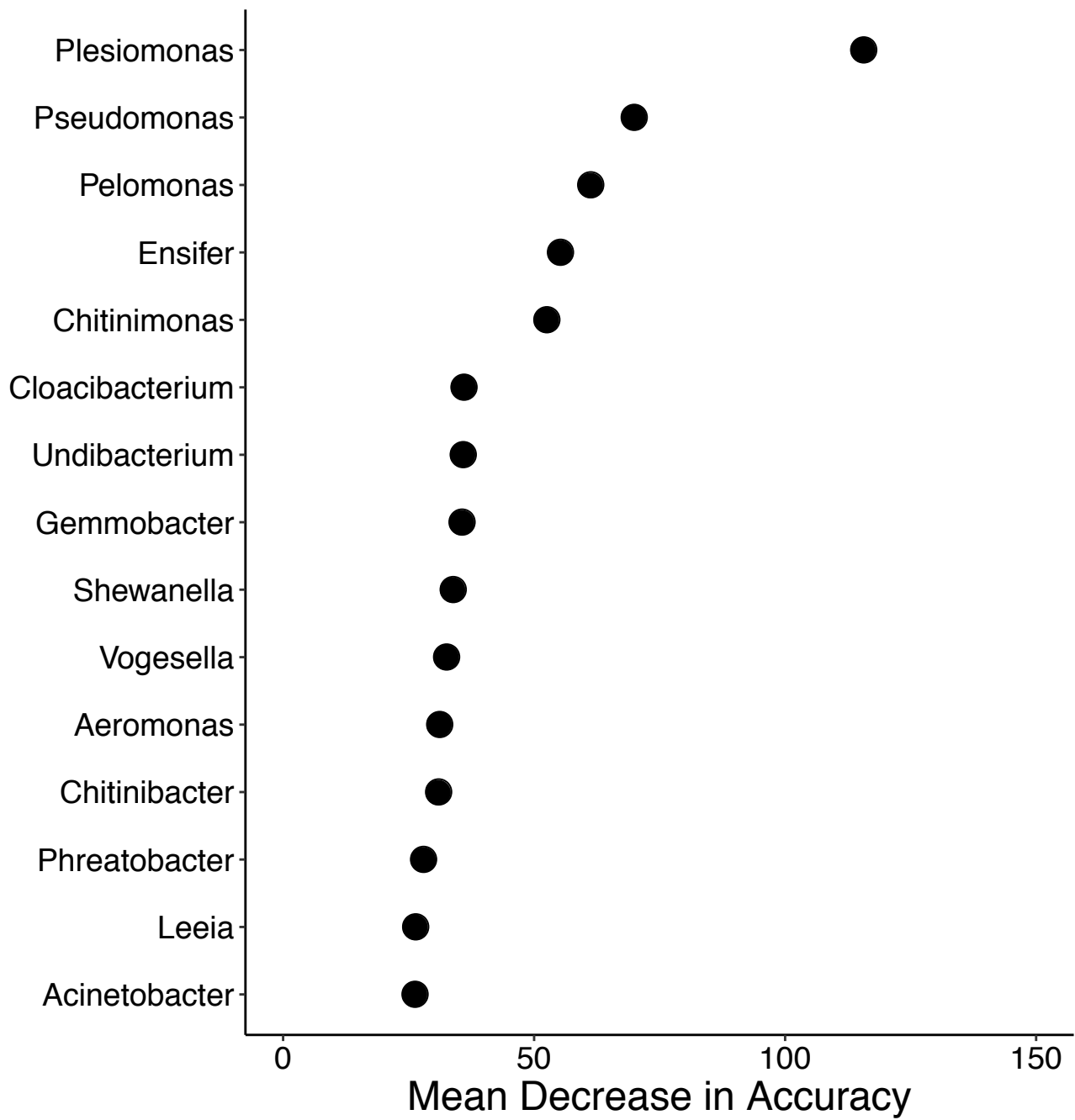

**Supplemental Figure 8**

Supplement: Supplementary file 17 — Figure S8. Random forest variable importance. A variable importance plot for the top 15 most important genera. Mean decrease in accuracy is scaled by variable standard deviation. (PDF 32 kb) [file 40168_2019_622_MOESM17_ESM.pdf]
