# Supplementary material for: A longitudinal assessment of host-microbe-parasite interactions resolves the zebrafish gut microbiome’s link to Pseudocapillaria tomentosa infection and pathology
Source: Microbiome. 2019 Jan 24;7:10. doi: 10.1186/s40168-019-0622-9 (PMC6346533; doi:10.1186/s40168-019-0622-9)
Supplement: Supplementary file 6 — Supplemental text. (DOCX 149 kb) [file 40168_2019_622_MOESM6_ESM.docx]

Supplemental methods:

***Pilot Parasite infection and burden quantification***

To create an infectious environment 30 *P. tomentosa* infected (donor) zebrafish were placed in an 80 L static flow tank for several weeks. Donor fish infection was confirmed by examining the feces for the presence of *P. tomentosa* eggs using light microscopy. Prior to the initiation of the experiment, these fish were sequestered in a net cage that was perforated such that feces from infected fish would pass into the tank below, thus maintaining an infectious environment while physically isolating these fish from the bottom portion of the tank. To infect fish, 65 *P. tomentosa* naïve adult 5D line zebrafish (recipient fish) were placed in the exposure tank for three days. After exposure the recipient fish were removed and randomly separated into six 3 L tanks (N = 10) and one additional 3 L tank (N = 5). Fish from these tanks were progressively evaluated at 6, 11, 18, 25, 32, 39 and 46 days post exposure (dpe.). Seventy-two hours before necropsy fish were isolated and individually housed in 1.5L tanks for fecal collection. Stool was collected from each tank every 24hrs during the 72hr period and the last sample (72hrs post isolation) was stored at -20˚C until processing. During the experiment, water conditions were maintained at: temperature 27.60±0.80˚C, pH 7.50±0.20, total ammonia 0.19±0.05 mg • L^-1^, and dissolved oxygen 6.59±0.29 mg • L^-1^. After the fecal collection the fish were euthanized by rapid chilling in iced water (2-4˚C) and the intestines were removed for parasitological analysis. Wet mounts were prepared from each intestine and examined with light microscopy to quantify the number of eggs, larvae, and adult worms present.

***Pilot 16S amplicon library preparation and sequencing***

Isolation of microbial DNA and 16S library preparation was conducted identically to the methods outlined in the main manuscript. A total of 200ng of amplicon library was pooled and the pooled library was then cleaned using the UltraClean^®^ PCR clean-up kit (MOBIO) and diluted to a concentration of 10nM. The final pooled and cleaned product was submitted to the Oregon State University Center for Genome Research and Biocomputing (CGRB) for cluster generation and 250bp paired end sequencing on an Illumina MiSeq instrument. This generated ~1.3 million 250bp paired end sequences. The forward reads were input into DADA2 for quality filtering, sequence variant calling using default parameters, and taxonomic assignment against the Silva reference database (v128). The resulting sequence variant table was rarefied to a depth of 5,000 counts for consistency with the main experiment.
