# Supplementary material for: A longitudinal assessment of host-microbe-parasite interactions resolves the zebrafish gut microbiome’s link to Pseudocapillaria tomentosa infection and pathology
Source: Microbiome. 2019 Jan 24;7:10. doi: 10.1186/s40168-019-0622-9 (PMC6346533; doi:10.1186/s40168-019-0622-9)
Supplement: Supplementary file 15 — Figure S7. Mycoplasma sequence variant abundance is elevated in exposed animals. Boxplots of A) Mycoplasma spp. Burns et al. clade members and B) the combined abundance of all Mycoplasma sequence variants. Red dots represent the abundance in animals that presented with neoplasms. (PDF 71 kb) [file 40168_2019_622_MOESM15_ESM.pdf]

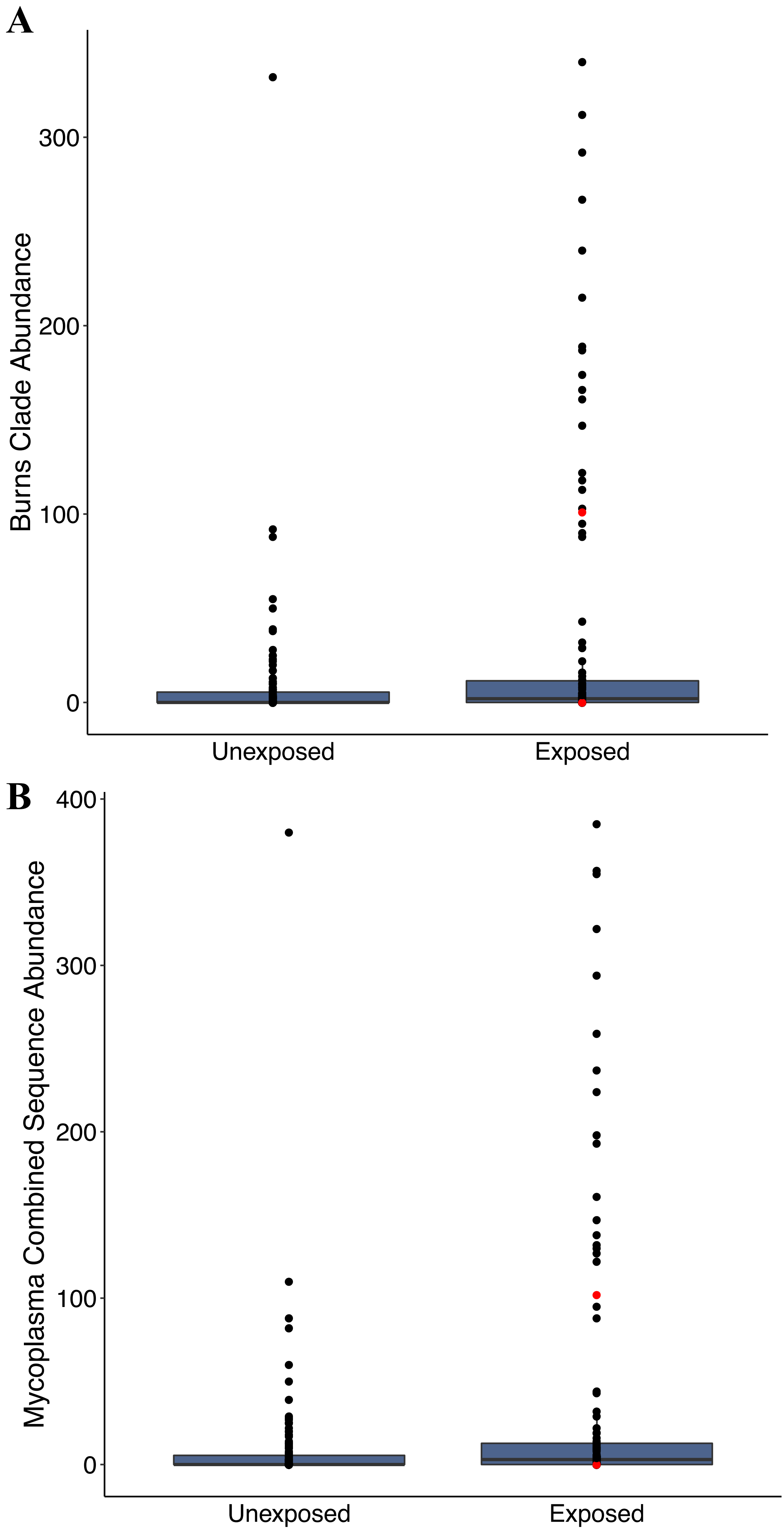

Supplemental Figure 7
